# Brain Evoked Response Qualification Using Multi-Set Consensus Clustering: Toward Single-Trial EEG Analysis

**DOI:** 10.1007/s10548-024-01074-y

**Published:** 2024-08-20

**Authors:** Reza Mahini, Guanghui Zhang, Tiina Parviainen, Rainer Düsing, Asoke K. Nandi, Fengyu Cong, Timo Hämäläinen

**Affiliations:** 1 Faculty of Information Technology, University of Jyväskylä, Jyväskylä, Finland; 2 Center for Mind and Brain, University of California -Davis, Davis, 95618 USA; 3 School of Biomedical Engineering, Faculty of Electronic and Electrical Engineering, Dalian University of Technology, Dalian, China; 4 Department of Psychology, Centre for Interdisciplinary Brain Research, University of Jyväskylä, Jyväskylä, Finland; 5 Department of Electronic and Electrical Engineering, Brunel University London, Uxbridge, UB8 3PH UK; 6 Department of Research Methods, Diagnostics and EvaluationInstitute of Psychology, University of Osnabrück, Osnabrück, Germany; 7 Key Laboratory of Integrated Circuit and Biomedical Electronic System, Dalian University of Technology, Dalian, 116024 China

**Keywords:** Single-trial EEG, Time window, Multi-set consensus clustering, Standardization, EEG/ERP microstates, Cognitive process

## Abstract

In event-related potential (ERP) analysis, it is commonly assumed that individual trials from a subject share similar properties and originate from comparable neural sources, allowing reliable interpretation of group-averages. Nevertheless, traditional group-level ERP analysis methods, including cluster analysis, often overlook critical information about individual subjects’ neural processes due to using fixed measurement intervals derived from averaging. We developed a multi-set consensus clustering pipeline to examine cognitive processes at the individual subject level. Initially, consensus clustering from diverse methods was applied to single-trial EEG epochs of individual subjects. Subsequently, a second level of consensus clustering was performed across the trials of each subject. A newly modified time window determination method was then employed to identify individual subjects’ ERP(s) of interest. We validated our method with simulated data for ERP components N2 and P3, and real data from a visual oddball task to confirm the P3 component. Our findings revealed that estimated time windows for individual subjects provide precise ERP identification compared to fixed time windows across all subjects. Additionally, Monte Carlo simulations with synthetic single-trial data demonstrated stable scores for the N2 and P3 components, confirming the reliability of our method. The proposed method enhances the examination of brain-evoked responses at the individual subject level by considering single-trial EEG data, thereby extracting mutual information relevant to the neural process. This approach offers a significant improvement over conventional ERP analysis, which relies on the averaging mechanism and fixed measurement interval.

**Supplementary Information:**

The online version contains supplementary material available at 10.1007/s10548-024-01074-y.

## Introduction

Electroencephalography (EEG) is a non-invasive neuroimaging technique that records electrophysiological brain activity using multiple electrodes placed on the scalp. For decades, cognitive neuroscience has utilized group-level averaging of EEG data to identify specific components of evoked activity that are associated with distinct cognitive functions. However, there is a growing need to qualify brain responses from individual subjects and single-trial EEGs, especially in clinical investigations. Due to the complexity and high noise in raw EEG data, averaging EEG trials—resulting in ERPs—has traditionally been used to study ERP components, which, in turn, are associated with specific perceptual, motor, or cognitive processes. Averaging is justified based on the assumption that single-trial EEG signals represent similar properties of the cognitive process in question, which can be identified through ERPs.

Although the ERP technique is popular due to its high signal-to-noise ratio (SNR), simplicity in statistical analysis, and interpretability of brain information processing via different ERP components, it does not fully capture potentially valuable information available in individual trials (Cohen and Cavanagh 2011; Delorme et al. 2002). Additionally, studying the variability of single trials is crucial in clinical studies due to the inhomogeneity among individual subjects (Knuth et al. 2006). This variability highlights the differences among participants within a single group and, notably, between distinct groups such as control and patient groups. The variability arises from two primary sources: the duration of the response and the variance between the means of response latencies. In contrast, ERP identifies the time-locked response to stimulus onset, reducing the contributions of physiological and recording noise that are not time-locked.

Various methods, including advanced statistical techniques, have been employed to investigate single-trial EEG data and extract ERP components. A significant portion of ERP studies have utilized independent component analysis (ICA; Makeig et al. 1997) and principal component analysis (PCA; Schölkopf et al. 1998) to extract shared ERP components from concatenated ERP data across all subjects (Bugli and Lambert 2007; Calhoun et al. 2009; Dien et al. 2007), as well as from single-trial EEG of individual subjects (Cong et al. 2010; Huster et al. 2020; Rissling et al. 2014; Zhang et al. 2023). Some studies have applied ICA to single-trial EEG (Delorme et al. 2002) with the objective of identifying brain responses by subjectively confirming the ERP component of interest. A key challenge for these methods is the variability in latency and phase across individual trials. Temporal PCA has been used to extract variable ERPs from single-trial EEG epochs, demonstrating subject-specific variations in the number of PCs associated with specific ERP components (Zhang et al. 2023). This suggests that the timing of neural responses (latency) and the brain oscillatory synchronization across brain regions (phase) differ across subjects. To mitigate trial inconsistency, some researchers have aligned brain responses within trials by adjusting stimulus and responses based on the averaged response and employed ICA decomposition for component selection (Jung et al. 2001; Onton et al. 2006).

Cluster analysis of EEG/ERP, as another objective approach, has gained attraction as a valuable tool for modeling event-related and resting-state EEG, aiming to isolate ERP components. The concept of EEG cluster analysis was first described by Lehmann et al. (Lehmann et al. 1987), introducing the ‘atom of thoughts’—quasi-stable electrical potentials (EEG microstates) that remain unchanged for brief periods, typically 80–100 ms (D’Croz-Baron et al. 2021). The cluster analysis of microstates involves two steps: calculating canonical cluster maps (template maps) that represent high explained variance, followed by reassigning these template maps to time points based on spatial correlation (Khanna et al. 2014). Two popular clustering techniques have been used in microstate analysis, modified *k*-means (Pascual-Marqui et al. 1995) and atomize and agglomerate hierarchical clustering (AAHC; Murray et al., 2008) on global field power (GFP)/GFP maxima points. However, microstate analysis disregards the polarity of the time point, which is substantial for ERP component analysis.

Various advanced clustering methods, such as the Gaussian mixture model for individual subjects (De Lucia et al. 2007b) and single-trial EEG (De Lucia et al. 2007a), as well as stimulus-related statistical information from single-trial responses (Tzovara et al. 2012b), have been employed in EEG analysis. Particularly, consensus clustering (Abu-Jamous et al. 2015; Liu et al. 2017) has demonstrated consistent and reliable outcomes for identifying ERPs from group-averaged ERP data (Mahini et al. 2020, 2022b). However, the low SNR and high degree of variety in single-trial EEG data present a challenge for clustering analysis, potentially leading to uncertain or erroneous results. Moreover, the extraction of ERPs from single-trial EEG for individual subjects remains underexplored in previous studies.

**Fig. 1:**
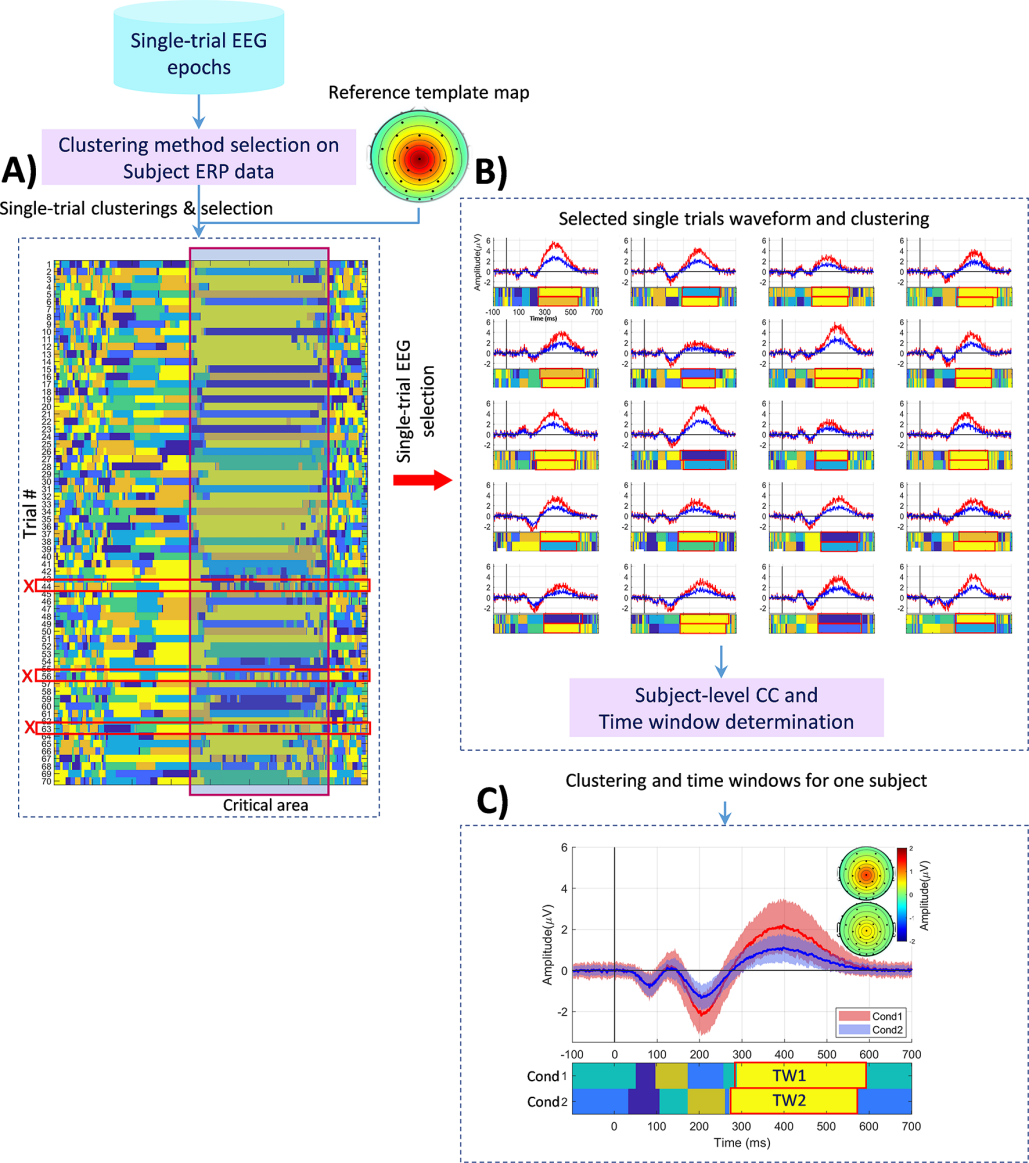
The proposed pipeline for identifying the ERP component shown in an individual subject using multi-trial consensus clustering. **A**) Selection of clustering methods for individual subjects based on ERP data and trial examination. Trials in the ‘critical area’ (i.e., selected based on the experimental mechanism for the expected ERP) are chosen, while trials with low or no correlation with the template map are discarded. **B**) Initiation of multi-set consensus clustering with the single-trial EEG epochs of the subject, followed by across-trials consensus clustering. **C**) Exploration for the optimal time window, examining inner similarity and spatial correlation of candidate maps. Abbreviations: Cond (condition), TW (time window), CC (consensus clustering)

This study aims to develop a robust method that effectively captures evoked responses for each condition/group at the individual subject level, introducing a multi-set consensus clustering-based pipeline (see Fig. 1). The pipeline begins by evaluating and selecting single trials based on spatial characteristics of obtained cluster maps compared to the elicited ERP components identified in group-averaged ERP data. Subsequently, the consensus clustering of single-trial EEG epochs aims to generate aggregated cluster maps from each trial, capturing the most relevant ERP responses. Second-level consensus clustering is then applied to identify consistent cluster maps across selected trials for each subject. A modified time window determination method is employed to explore the latency of the target ERP precisely at the individual subject level. We used simulated and real EEG data to assess the proposed pipeline’s efficacy. The goal is to develop a robust method that effectively captures evoked responses for each condition/group at the individual subject level. Ultimately, this approach aims to reliably identify consistent ERP components within the single-trial EEG data of individual subjects.

## Materials and Methods

### Simulated EEG Data

EEG data were simulated using the SEREEGA MATLAB toolbox (Krol et al. 2018), incorporating four pre-defined ERP components: N1, P2, N2, and P3. Data were simulated for two conditions and 20 subjects, with 70 trials per condition, using 32 simulated scalp electrodes. The ERP components were first generated as ground truth with defined latency, amplitude, and width, and then random variations in amplitude, width, and duration were applied to generate individual subjects’ data. Additional white Gaussian noise (e.g., 1 µV) was added to the EEG signals. White noise was used in our simulations due to its flat power spectral density, simplifying initial signal processing and providing a baseline for testing the method (Niedermeyer and da Silva 2005). The EEG signals were epoched from − 100 to 700 ms, and the sampling rate was set at 500 Hz (i.e., each EEG epoch had 400 time points) to expedite processing.

**Fig. 2:**
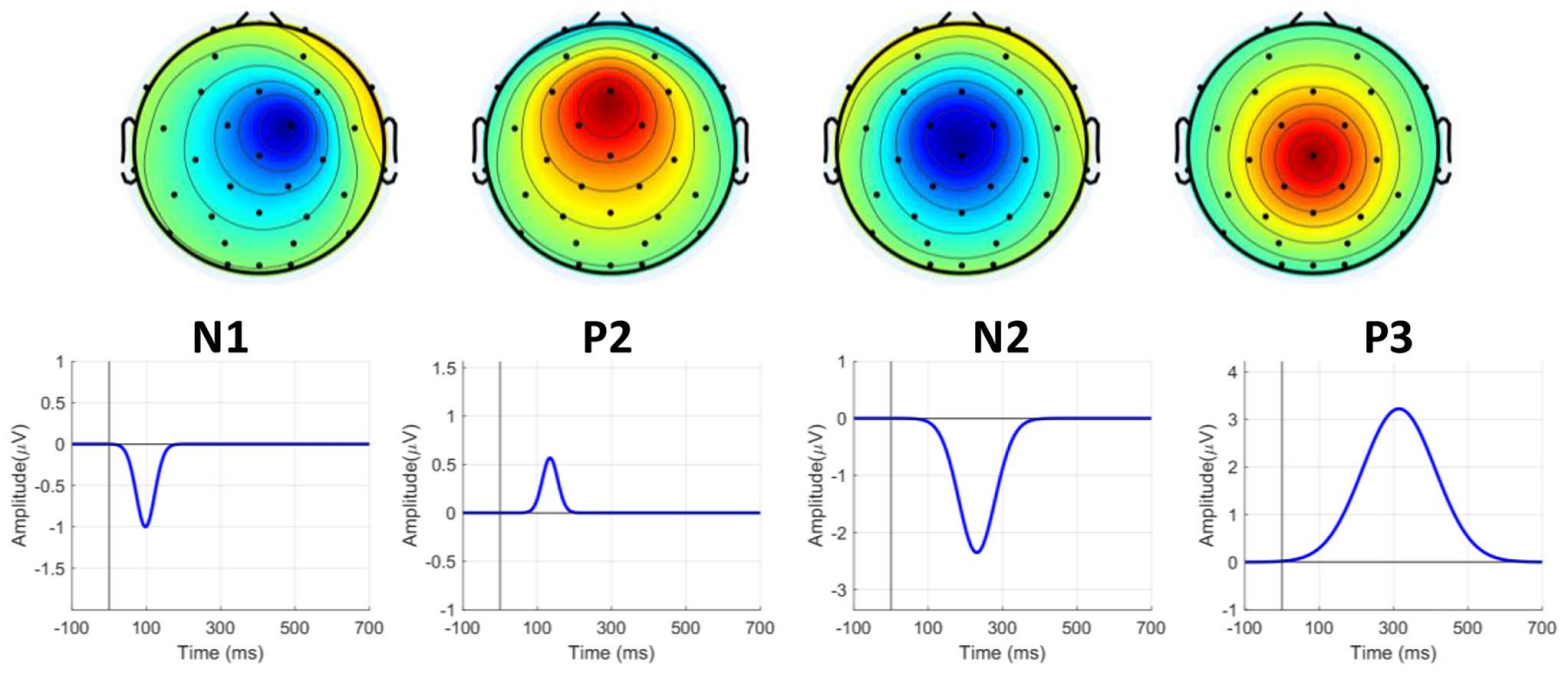
Illustration of the topographical configuration and temporal properties of four pre-defined ERP components: N, P2, N2, and P3

Significant effects were mathematically incorporated into the P2, N2, and P3 components. The N2 and P3 components were examined as examples of negative and positive polarity ERP components, respectively. More specifically, for subjects’ data, the N2 component, characterized by a negative amplitude, was generated with random latencies between 200 and 250 ms, durations of 100 to 200 ms, and magnitudes of -2.5 to -1.5 µV. Similarly, the P3 component, characterized by a large positive amplitude, was generated with random latencies between 250 and 450 ms, 350 to 500 ms durations, and 4 to 6 µV magnitudes. Additionally, for EEG epochs, random deviations of 50 ms in latency, 2 µV in amplitude, and 50 ms in duration were applied for the N2 component, and random deviations of 100 ms in latency, 3 µV in amplitude, and 100 ms in duration were applied for the P3 component. Finally, the electrode sites of interest for the N2 and P3 components were Fz and Cz, respectively. Figure 2 demonstrates the spatial and temporal properties of the pre-defined ERP components.

### Real EEG Data

Real EEG data from a previous study (Kappenman et al. 2021) from an active visual oddball task was used for assessment. The P3 component, originally designed to assess ‘stimulus evaluation times,’ focuses on response time duration rather than the component’s latency (Luck et al. 2009). In the prior study (Kappenman et al. 2021), letter stimuli (A, B, C, D, and E) were used, with one letter designated as the target and the others serving as nontargets. The P3 component was defined as the maximum positive peak occurring around 300 to 600 ms, which served as the critical area for the ERP component in this paper.

EEG data were recorded from 40 participants (25 female and 15 male) using 30 scalp electrodes according to the international 10/20 system in two conditions: ‘Rare’ and ‘Frequent.’ The recorded signals were digitized at a resolution of 1024 Hz, then downsampled to 256 Hz for faster processing, and referenced offline to the average of P9 and P10. Approximately 50 to 70 trials for each subject’s condition were selected in the prior study, with fewer trials in some cases. Epochs were selected from 200 ms before the stimulus onset to 800 ms after the stimulus onset. DC noise was removed, and high-pass and low-pass filters were meticulously applied at 0.1 and 20 Hz, respectively, to minimize any influence on stimulus onset latency. ICA was subsequently applied to address component-related artifacts, including eyeblinks and eye movements, which were removed via visual inspection and topographic representation of the components. Statistical power analysis was performed on the Pz electrode (as recommended by the experimenters) and the selected trials (see Sect. “Trial Selection”).

### Proposed Method

This section details each stage of our proposed pipeline, as depicted in Fig. 1. This pipeline is designed to identify the event-related potential (ERP) of interest for individual subjects through three main steps: trial selection, multi-set consensus clustering, and time window determination of ERP. Additionally, to facilitate further research, we have made the proposed pipeline’s simulated data and demo code available on GitHub at the following link: https://github.com/remahini/Single_trial_EEG_MSCC.

#### Trial Selection

Each trial was examined to eliminate those with low or no correlation to the pre-defined (in the simulated data) or identified component from the group average ERP data, referred to as the template map. To achieve this, each trial was clustered using consensus clustering, and the resulting cluster maps were assessed for the presence of the specific ERP component via spatial correlation comparison. The ERP template map’s topographical configuration was used to mask clustering results by measuring the spatial correlation (Murray et al., 2008) between candidate cluster maps—those with high inner similarity within the expected experimental interval—and the ERP template map. The inner similarity is defined as the Pearson correlation coefficient between any two time points $\:i$ and $\:j$ where $\:i\ne\:j$.

Following the microstates analysis for EEG/ERP, we have used the Pearson cross-correlation coefficient for calculating the spatial correlation (Koenig et al. 2008; Murray et al., 2008), which can be defined for two time points as:

1 $$Cor{r_{u,v}} = {{\mathop \sum \nolimits_{i = 1}^F {u_i} \cdot {v_i}} \over {||u|| \cdot ||v||}},$$

where,

$$|u|| = \sqrt {\mathop \sum \limits_{i = 1}^F {u_i}^2} \,,\,\,||v|| = \sqrt {\mathop \sum \limits_{i = 1}^F {v_i}^2} .$$

Here, $\:F$ is the number of electrodes, and $\:u$ and $\:v$ are the topographical maps of the two time points. The mean or centroid of the topographies within the cluster map’s duration is used when comparing cluster maps.

For cluster analysis, each single-trial EEG epoch was treated as a dataset for clustering, with time points as observations and electrodes as features (e.g., dataset size: 256 time points × 28 electrodes). Section ’“Multi-Set Consensus Clustering” details the clustering design. Two sensitivity parameters controlled trial examination: inner similarity (e.g., > 0.90) and spatial correlation (e.g., > 0.50 with the template map). These parameters could be adjusted if no map was found. Therefore, the proposed method aimed to retain at least 50% of trials per subject and condition by decrementing the spatial correlation threshold from 0.70 to 0.50, depending on the data, to ensure sufficient trials for reasonable analysis.

#### Multi-Set Consensus Clustering

The consensus clustering method was designed using clustering methods implemented in our toolbox (Mahini et al. 2022b) and was applied at two levels: individual trial clustering and ensemble clustering across the trial results for each subject/condition. This two-step procedure is referred to as multi-set consensus clustering in this context. Before cluster analysis, a pre-clustering method selection step was implemented using the M-N plot method (Abu-Jamous et al. 2014; Mahini et al. 2022b) on each subject’s temporally concatenated ERP dataset to select appropriate clustering methods for feeding consensus clustering. Two criteria were used: the inner similarity of samples (threshold, e.g., > 95) and the duration of the identified ERP (threshold, e.g., > 50 ms).

Aside from that, while estimating the optimal number of clusters from individual subject ERP data could be more precise, we determined the optimal number of clusters (Mahini et al. 2022b) by testing the inner similarity of the estimated time window from the group average ERP data to maintain simplicity. This approach to determining the optimal number of clusters examines a range of clusters, for example, from 2 to 15, seeking where the inner similarity of the estimated time windows is stable and high (e.g., > 0.95). Selected trials were then clustered using consensus clustering. We used the cluster-based similarity partitioning algorithm (CSPA) consensus function (Karypis and Kumar 1998; Nguyen and Caruana 2007), which was chosen based on hypergraph partitioning, using the ‘supra’ test (Ghosh et al. 2002) to find the best ensemble clustering solution for trial and subject-level consensus clustering. Using CSPA allows for some tolerance of variations in information distribution across single trials.

Let us consider the consensus clustering problem for dataset X={x^1^,x^2^,...,x^n^ }, with $\:n$ samples into $\:K$ groups, where each group is represented by a centroid $\:{\mu\:}_{k}$, $\:k=\left\{\text{1,2},\:\dots\:,\:K\right\}$. Each sample $\:{x}_{t}\in\:\:{R}^{F}$, $\:t=\{\text{1,2},\dots\:,n\}$ and $\:F$ denotes the number of features (electrodes in the EEG scalp). A set of $\:m\:$clusterings $\:{L}^{(\text{1,2},\:\dots\:,\:m)}$ is used for combining clusterings into a final clustering $\:L$. The objective function for cluster ensemble from $\:m\:$clusterings can be defined as $\:\varGamma\:$ : $\:{N}^{n\times\:m}\to\:{N}^{n}$, which maps the clusterings to a set of clusters.

2 $$\:\varGamma\::\left\{{L}^{\left(i\right)}\right|i\in\:\{\text{1,2},\dots\:,m\}\}\to\:L,$$

thus, given a set of clusterings $\:\left\{{L}^{\left(i\right)}\right|i\in\:\{\text{1,2},\dots\:,m\}\},$ the goal is to explore the firmest clustering that shares the most information from all clusterings. Therefore, the optimal clustering from $\:m$ clusterings can be defined as:

3 $$\:{{L}^{*}}_{tm}={argmax}_{L\in\:L}\:{\sum\:}_{l=1}^{m}{\varGamma\:}^{\left(NMI\right)}\left({L}_{l}\right),$$

where $\:\varGamma\:$ denotes a similarity measurement, NMI (Meila, 2007), which measures mutual information between a set of $\:m$ clusterings. $\:{{L}^{*}}_{tm}$ is the optimally combined clustering with maximum average similarity to all other clusterings $\:{L}_{l}$ for the individual trial.

Next, we combine the clustering results of trials using further trial-level consensus clustering. The consensus function across the trials can be presented as follows:

4 $${L^{**}}_c^p = argma{x_{L \in {L_T}}}\mathop \sum \limits_{i = 1}^{T_c^p} \Gamma \left( {L_i^{{\rm{}}}} \right),$$

where, $$\:{T}_{c}^{p}$$ denotes the number of selected trials for subject *p* in condition *c.*$$\:{{L}^{**}}_{c}^{p}$$ denotes the result of consensus clustering across the trials. These two steps—clustering of each trial and across the trials—are collectively called multi-set consensus clustering, and for each subject $$\:p$$ can be noted by:

5 $$\:{{L}^{**}}_{c}^{p}={argmax}_{L\in\:{L}_{X,T}}{\sum\:}_{i=1}^{{T}_{c}^{p}}{\sum\:}_{j=1}^{{R}_{p}}\varGamma\:\left({L}_{j}^{i}\right).$$

Here, $\:{L}_{j}^{i}$​ represents all clusterings for the $\:{i}^{th}$ subject’s trials under condition $\:c$, using the $\:{j}^{th}$ set of clustering methods from $\:{R}_{p}$ (i.e., the subject’s selected clustering methods).

Given the CSPA consensus function’s mechanism of aggregating the most consistent cluster sets from diverse input clusterings, this approach ensures that consecutive time points are assigned to a cluster map sharing similar information across most cluster sets.

#### Time Window Determination

Once clustering results were obtained from the individual subjects, a modified version of the time window determination was applied for each subject. The time window determination (Mahini et al. 2020) was modified through two criteria in two steps. First, candidate cluster maps with high inner similarity (e.g., > 0.95) were detected within the experimentally interesting interval. Experimental parameters, including expected response latency, estimated duration, and region of interest, were derived from prior studies (Kappenman and Luck 2012). Next, among the selected candidate cluster maps, those with a better fit and higher spatial correlation with the template map of the interesting ERP (e.g., > 0.90, adjustable if needed) were chosen. It is important to note that time window determination was used at the trial level to calculate statistical scores (see Sect. “Performance Analysis and Reproducibility Test”) and at the subject level to identify ERP components from the clustering results.

### Performance Analysis and Reproducibility Test

We designed a reproducibility assessment method encompassing both experimental and signal processing evaluations. To this aim, a Monte Carlo test was implemented on the trials’ clustering, testing the reliability of consensus clustering on single-trial EEG to quantify (scoring) the ERP of interest, which can be used in similar signal processing methods. This method ensures high reproducibility and stability, making it valuable for the community in hypothesis testing. The primary goal of this study is to develop a robust clustering analysis for identifying specific cognitive processes of individual subjects.

#### Inter-Trial and Inter-Subject Reproducibility Tests

Inter-trial and inter-subject reproducibility measure the consistency and predictability of stimulus-locked response properties at the individual trial and subject levels. Unlike repeatability, which assesses the consistency of repeated results, reproducibility evaluates consistent results from different sources (e.g., trials, subjects) that are not identical. In this context, reproducibility refers to the consistency of scores calculated using the proposed pipeline. This concept, inspired by the standard measurement error ($\:SME$) introduced by Luck et al. (Luck et al. 2021) for ERP, evaluates the quality of scores and data measurement. Here, scoring refers to the estimated component’s properties, such as time window properties, mean amplitude at the electrode site, spatial correlation, and inner similarity obtained from individual subjects/trials. Two evaluation methods—analytical and Monte Carlo-based measurements—were used to assess the identified ERP components.

For analytical scores, we calculated the standard error ($\:SE$) of estimated scores at two levels: single-trial EEG and individual subject ERP. Generally, the estimated $\:\widehat{SE}$ from $\:n$ results in a given score item can be calculated as:

6 $$\:\widehat{SE}=\:\frac{\widehat{SD}}{\sqrt{n}}\:,$$

where the $\:\widehat{SD}$ is the estimated standard deviation (SD) of the scores, and $\:n$ is the number of contributed scores. Note that the true value of SE is unknown; thus, its estimation is denoted as $\:\widehat{SE}$ in the subsequent sections. Leveraging that, given $\:n$ selected trials of one condition and calculated scores from each trial, the standard error can be calculated from Eq. 6. Score items used in our measurement process include: (i) at the single-trial EEG level, spatial correlation is assessed between the estimated ERP of trials and the template map from the mean topography in the determined time window. For example, the result of $\:\widehat{SE}$ across the spatial correlation scores reflects the spatial error at the individual trial level. Similarly, the temporal reproducibility is evaluated by examining the consistency of estimated time windows across trials. (ii) At the individual subject level, the reproducibility of spatial and temporal properties of estimated time windows is evaluated for qualifying ERP.

To evaluate the proposed method, a Monte Carlo test was conducted, assuming sufficient scorers for hypothesis testing. Details of the Monte Carlo procedure are described in the following subsection.

#### Monte Carlo and Reliability Tests

A Monte Carlo test was established by creating a pool of selected trials’ clusterings for each condition and regenerating the same number of trials for each subject *with replacement*. Consensus clustering was then performed across the generated trials’ clusterings from each iteration of the Monte Carlo procedure, repeated 1000 times to calculate the scores and test their reproducibility. The test was specifically designed for simulated data with a significant effect size for the ERP components, namely the N2 and P3 components. Thus, the null hypothesis tests the absence of an effect size while repeating the pipeline from generated trials of simulated subjects in the iterations. Notably, trials/results can be simulated by generating an adequate number of trials multiple times for each condition and subject rather than repeating the experiment many times.

Hence, given $\:R$ repeats of the selection procedure and scores, $\:{\widehat{mcSE}}_{s}^{c}$, i.e., the estimated standard error of the Monte Carlo for subject $\:s=\{\text{1,2},\:\dots\:,\:S\}$, is calculated as averaged squared errors as:

7 $$\:\widehat{{mcSE}_{s}^{c}}=\sqrt{\frac{{\sum\:}_{r=1}^{R}{\widehat{S{E}_{r}}}^{2}}{R}},$$

where the standard error ($\:{\widehat{SE}}_{r}$) for each of the repeats $\:r=\left\{\text{1,2},\:\dots\:,\:R\right\}$ is calculated as:

8 $$\:\widehat{{SE}_{r}}=\:\frac{\widehat{{SD}_{r}}}{\sqrt{{N}_{c}^{s}}}\:,$$

and $\:{N}_{c}^{s}$ denotes the number of trials for subject $\:s$ in condition $\:c$ for each iteration. Therefore, the scores from each generation can be calculated followed by obtaining the measurement error for all the individual subjects as aggregated error:

9 $$\:MS\left(\widehat{SE}\right)=\frac{\widehat{{SE}_{1}^{2}}+\:\widehat{{SE}_{2}^{2}}+\dots\:+\widehat{\:{SE}_{S}^{2}}}{S}.$$

Furthermore, an additional parameter called total error $\:{\widehat{Var}}_{all}$ is calculated from the individual subjects $\:{\widehat{Var}}_{par}$ called true variance, and the measurement error (calculated from Eq. 8). This calculation can be illustrated as:

10 $$\:{\widehat{Var}}_{all}=\:{\widehat{Var}}_{par}+MS\left(\widehat{SE}\right),$$

Although this metric was not originally designed for single-trial EEG analysis, we adapted it to generate simulated clusterings obtained from individual trials during the Monte Carlo test. This adaptation assumes that sufficient trials are available for consensus clustering. The clustering generation procedure reduces the complexity of applying consensus clustering since no generation step (clustering) is required in the trials. Consequently, we seamlessly integrated the scoring results of the trials with individual subject scores, ensuring robust evaluations. Therefore, the reliability of the measurement can be calculated as follows:

11 $$\:\widehat{Reliability}=\:1-\:\frac{MS\left(\widehat{SE}\right)}{{\widehat{Var}}_{all}}.$$

Furthermore, we used Cronbach’s alpha and standard error of measurement (SEM) to calculate the reliability, estimating the error in individual scores within the subjects. The Cronbach’s alpha is calculated as:

12 $$\:\alpha\:=\:\frac{q}{q-1}(1-\:\frac{{\sum\:}_{i=1}^{q}{\widehat{V}}_{i}}{{\widehat{V}}_{tot}}),$$

where, $\:q$ is the number of items (the number of scoring tests), $\:{\widehat{V}}_{i}$ denotes the variance associated with each measure, and $\:{\widehat{V}}_{tot}$ is the variance associated with all the scores. The $\:\widehat{SEM}$ is then calculated as:

13 $$\:\widehat{SEM}=\widehat{SD}\:\times\:\:\sqrt{1-\alpha\:}$$

#### Statistical Analysis

Repeated measures statistical analyses of variances (ANOVA) was conducted to assess the null hypothesis where there was no significant difference between conditions for both pre-defined ERP components in the simulated data. The within-subject factor was condition assessed at the Fz electrode site for the N2 component and the Cz site for the P3 component. For the real data, repeated measures ANOVA was performed with a within-subject factor of stimulus (conditions: ‘Rare’ and ‘Frequent’) at the Pz electrode site, matching the original study’s focus. The null hypothesis tested was that there is no significant difference between conditions in the determined time windows from individual subjects. The mean amplitude was calculated within these estimated time windows to investigate the effect of the stimulus on the P3 component. Statistical comparisons were made at an alpha level of 0.05.

## Results

Here, we present the clustering outcomes and the spatial-temporal characteristics of the identified ERPs for individual subjects in both simulated and real datasets. Additionally, we conduct an in-depth performance analysis and present reproducibility results.

### Multi-Set Consensus Clustering Results and Temporal Properties

Two series of consensus clustering were performed. Firstly, consensus clustering on group average ERP data aimed to identify the P3 component (used as the reference) in the real data. Secondly, multi-set consensus clustering was applied to single-trial EEG data in simulated and real datasets. The pre-defined ERP components served as ground truth in the simulated data. The optimal number of clusters determined was 6 for simulated and real data from the group average ERP data.

#### Clustering Results for the Simulated Data

**Fig. 3:**
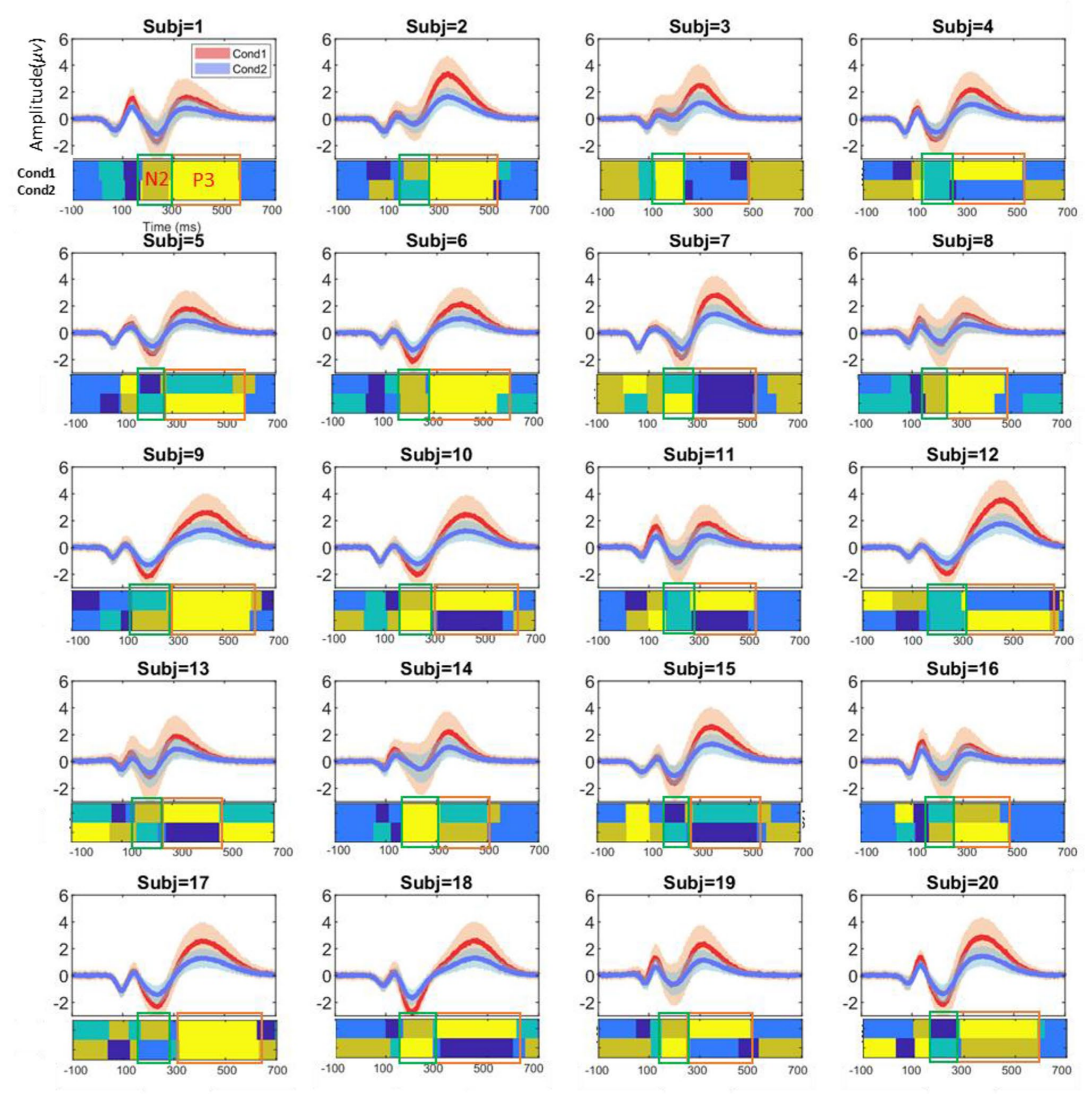
The obtained clustering results, with colored areas representing cluster maps, using multi-set consensus clustering on the original subjects’ ERP waveforms (in Cz electrode) from two conditions. Clustering was applied in six clusters as the optimal number of clusters based on the group’s average ERP data. The colored rectangles denote the corresponding time windows of N2 (indicated in green) and P3 (indicated in red) for ‘Cond1’ and ‘Cond2’, respectively. Abbreviations: Cond1(condition 1) and Cond2 (condition 2)

The set of clustering methods identified from the M-N plot examination on the ERP data of each subject (see Sect. Multi-Set Consensus Clustering) was applied to single trials of each subject. Table 1 illustrates the selected clustering methods for each subject. Figure 3 presents the clustering results, displaying ERP waveforms at the Cz electrode with estimated time windows for N2 and P3 components (highlighted in blue and red, respectively). Detailed information regarding the identified N2 and P3 components for individual subjects is provided in Tables 2 and 3. The analysis of these results, including cluster analysis and ERP determination, reveals reasonable consistency in determined time windows and spatial correlations, although with noticeable variability across conditions and subjects.

**Table 1:** Selected clustering methods for individual subjects’ data in the simulated data. The examination of the clustering method was performed via the M-N plot test (Mahini et al., 2022a). The replacement list was employed if no suitable method was found or if an individual method was selected. The number of clusters was determined to be six (the optimal number of clusters)

Subj_ID	Selected methods	Replacement List
S1	KM, HC, MKM, KMD, GMM	-
S2	KM, HC, SOM, DSPC, SPC, KMD	-
S3	One method (KM)	KM, HC, MKM, SPC, KMD, GMM
S4	KM, HC, FCM, SOM, DSPC, MKM, KMD, GMM	-
S5	HC, FCM, DSPC, MKM, SPC, KMD, GMM	-
S6	KM, HC, SOM, MKM, SPC, KMD, GMM	-
S7	KM, FCM, SOM, SPC, KMD, GMM	-
S8	KM, HC, FCM, DSPC, MKM, SPC, GMM	-
S9	KM, HC, FCM, SOM, MKM, SPC, KMD, GMM	-
S10	HC, FCM, MKM, SPC, KMD, GMM	-
S11	KM, HC, FCM, SOM, DSPC, SPC, KMD, GMM	-
S12	KM, HC, FCM, SOM, DSPC, MKM, SPC, KMD, GMM	-
S13	KM, HC, SOM, DSPC, MKM, KMD, GMM	-
S14	KM, HC, FCM, SOM, DSPC, MKM, SPC, KMD, GMM	-
S15	KM, HC, DSPC, MKM, SPC, KMD, GMM	-
S16	KM, HC, FCM, DSPC, MKM, SPC, KMD, GMM	-
S17	KM, HC, DSPC, MKM, SPC, KMD, GMM	-
S18	KM, FCM,DSPC, MKM, KMD, GMM	-
S19	KM, HC, FCM, SOM, DSPC, MKM, SPC, KMD, GMM	-
S20	KM, HC, FCM, DSPC, MKM, SPC, KMD, GMM	-

*Abbreviations* KM (*k*-means), HC (hierarchical clustering), SOM (self-organizing map), DSPC (diffusion map spectral clustering), MKMS (modified *k*-means), SPC (spectral clustering), KMD (*k*-medoids clustering), and GMM (Gaussian mixture model)

**Table 2:** Average scores from 100 repetitions for the time window (start, end), inner similarity, mean amplitude (at Fz site), and spatial correlation of estimated N2 components in the simulated data

	Score_Cond1										Score_Cond2									
Subj_ID	TW		TW		Innsim/SD		Amp(µv)/SD		Corr/SD		TW start(ms)/SD		TW end(ms)/SD		Innsim/SD		Amp(µv)/SD		Corr/SD	
	start(ms)/SD		end(ms)/SD																	
S1	173.2	5.02	284	8.6	0.85	0.17	-0.71	0.07	1	0	171.6	2.19	289.6	4.1	0.87	0.08	-0.45	0.04	1	0
S2	164.8	8.2	256.4	3.29	0.84	0.13	-0.7	0.14	0.82	0.05	168	4	260.4	5.37	0.87	0.06	-0.45	0.05	0.93	0.02
S3	162.86	5.05	279.2	9.65	0.92	0.06	-0.71	0.09	1	0.01	161.26	5.52	278.2	9.85	0.88	0.11	-0.43	0.05	0.95	0
S4	152	0	247.6	8.17	0.93	0.08	-0.73	0.08	1	0	167.6	1.79	252.4	4.77	0.94	0.04	-0.45	0.06	1	0
S5	166.4	3.29	259.6	10.62	0.95	0.04	-0.69	0.16	0.99	0	169.6	2.61	268	9.7	0.95	0.03	-0.45	0.05	1	0
S6	166.4	5.18	272.8	4.82	0.92	0.05	-0.69	0.14	0.97	0	163.6	3.29	274.8	7.56	0.93	0.06	-0.45	0.06	0.99	0
S7	159.2	9.12	274.4	7.4	0.85	0.13	-0.7	0.08	1	0	161.2	6.54	273.6	2.61	0.96	0.07	-0.44	0.05	1	0
S8	164.34	11.09	296.8	10.27	0.8	0.18	-0.69	0.13	0.98	0.11	162.86	4.38	267.6	11.44	0.81	0.06	-0.46	0.05	0.98	0.01
S9	145.6	4.77	266.8	3.9	0.98	0.02	-0.71	0.1	0.97	0	162.74	10.04	276.4	3.85	0.94	0.04	-0.45	0.05	0.99	0
S10	163.2	1.79	292	4	0.97	0.03	-0.7	0.09	0.98	0	164.4	2.19	291.6	2.61	0.99	0.01	-0.44	0.04	0.99	0
S11	170.8	3.03	264	6.78	0.86	0.09	-0.71	0.08	0.99	0.01	166	2.45	263.2	4.6	0.93	0.02	-0.45	0.05	1	0
S12	170.4	2.19	297.2	2.28	0.99	0	-0.71	0.08	0.98	0	167.2	3.63	303.2	3.03	0.98	0.01	-0.45	0.05	0.99	0
S13	165.28	15.62	273.74	11.9	0.86	0.14	-0.69	0.13	0.97	0.09	163.14	5.25	304.4	9.71	0.89	0.13	-0.45	0.05	1	0
S14	167.2	7.16	303.2	6.42	0.82	0.06	-0.69	0.14	0.98	0.06	173.2	5.22	294	9.59	0.84	0.05	-0.45	0.05	0.95	0.01
S15	157.6	2.97	247.2	5.4	0.95	0.02	-0.71	0.08	1	0	157.2	5.22	251.6	7.54	0.94	0.06	-0.45	0.04	1	0
S16	164.84	15.68	271.84	10.55	0.89	0.08	-0.71	0.13	0.97	0.12	162.74	2.83	276	11.05	0.9	0.1	-0.45	0.05	1	0
S17	168	3.46	296.4	3.58	0.97	0.02	-0.7	0.11	0.98	0	167.2	4.6	306.4	6.07	0.93	0.03	-0.45	0.06	0.99	0
S18	164.4	5.37	277.2	4.6	0.98	0.02	-0.71	0.08	0.96	0	162.4	6.69	277.6	5.73	0.97	0.02	-0.45	0.05	0.98	0
S19	164.34	14.7	272.96	23.69	0.9	0.09	-0.7	0.15	0.97	0.14	162.44	1.1	252.4	5.37	0.88	0.07	-0.45	0.05	0.96	0.01
S20	167.2	1.79	269.6	2.19	0.98	0.01	-0.71	0.09	0.99	0.01	168.8	3.35	271.2	5.76	0.98	0.02	-0.45	0.05	1	0
**Mean**	**163.9**	**6.27**	**275.15**	**7.41**	**0.91**	**0.07**	**-0.7**	**0.11**	**0.97**	**0.03**	**165.16**	**4.14**	**276.63**	**6.51**	**0.92**	**0.05**	**-0.45**	**0.05**	**0.98**	**0**
**SD**	**6.21**	**4.61**	**15.75**	**4.74**	**0.06**	**0.05**	**0.01**	**0.03**	**0.04**	**0.05**	**3.84**	**2.05**	**16.59**	**2.78**	**0.05**	**0.03**	**0.01**	**0**	**0.02**	**0**

*Abbreviations* Cond1 (condition 1), Cond2 (condition 2), TW (time window), Subj (subject), Innsim (inner similarity), Amp (amplitude), and Corr (spatial correlation

**Table 3:** Average scores from 100 repetitions for the time window (start, end), inner similarity, mean amplitude (at Cz site), and spatial correlation of estimated P3 components in the simulated data

	Score_Cond1										Score_Cond2										
Subj_ID	TW_start(ms)/SD		TW_end(ms)/SD		Innsim/SD		Amp(µv)/SD		Corr/SD		TW_start(ms)/SD		TW_end(ms)/SD		Innsim/SD		Amp(µv)/SD		Corr/SD		
S1	286.40	5.90	559.60	40.56	0.93	0.05	1.34	0.14	1.00	0.00	297.20	7.01	544.40	24.96	0.98	0.03	0.64	0.07	1.00	0.00	
S2	271.20	8.90	526.40	15.65	0.99	0.01	1.29	0.16	1.00	0.00	272.80	4.60	526.00	14.14	0.99	0.01	0.64	0.09	1.00	0.00	
S3	248.40	14.72	442.00	28.64	0.98	0.01	1.30	0.16	1.00	0.00	254.00	10.95	448.40	18.73	0.98	0.01	0.64	0.07	1.00	0.00	
S4	257.60	4.56	525.20	8.79	0.97	0.01	1.31	0.14	1.00	0.00	261.60	6.99	517.60	25.23	0.96	0.03	0.64	0.08	1.00	0.00	
S5	275.20	7.56	563.20	21.94	0.97	0.04	1.31	0.16	1.00	0.00	274.00	11.92	562.80	38.82	0.89	0.09	0.64	0.09	1.00	0.00	
S6	290.80	8.56	567.60	8.88	1.00	0.00	1.33	0.16	1.00	0.00	290.80	10.55	553.60	32.91	0.98	0.04	0.63	0.08	1.00	0.00	
S7	284.00	6.48	560.80	4.38	0.98	0.02	1.30	0.15	1.00	0.00	292.40	5.18	540.80	24.02	0.99	0.01	0.63	0.06	1.00	0.00	
S8	268.00	15.94	453.20	35.17	0.96	0.05	1.34	0.15	1.00	0.00	274.00	15.94	459.60	20.12	0.96	0.02	0.64	0.08	1.00	0.00	
S9	295.20	5.22	609.20	23.22	1.00	0.00	1.29	0.14	1.00	0.00	300.40	7.80	593.20	28.62	1.00	0.00	0.63	0.08	1.00	0.00	
S10	315.60	6.99	589.20	15.01	1.00	0.00	1.35	0.16	1.00	0.00	310.00	5.48	608.40	5.37	0.99	0.01	0.63	0.08	1.00	0.00	
S11	264.80	4.15	510.00	14.56	0.96	0.02	1.32	0.16	1.00	0.00	267.20	4.60	495.20	7.56	0.93	0.03	0.64	0.08	1.00	0.00	
S12	321.60	3.29	644.40	11.17	1.00	0.00	1.31	0.17	1.00	0.00	323.20	3.03	638.00	10.68	1.00	0.00	0.64	0.08	1.00	0.00	
S13	274.00	6.78	466.40	18.46	1.00	0.00	1.29	0.16	1.00	0.00	268.00	4.69	466.40	26.13	0.98	0.02	0.65	0.08	1.00	0.00	
S14	309.20	9.23	493.20	7.43	0.99	0.01	1.32	0.16	1.00	0.00	304.00	9.06	464.00	15.62	0.97	0.02	0.63	0.08	1.00	0.00	
S15	264.80	7.29	543.60	13.89	0.98	0.03	1.30	0.14	1.00	0.00	272.40	3.58	518.00	19.34	0.99	0.01	0.64	0.07	1.00	0.00	
S16	271.20	9.96	433.60	33.54	0.87	0.08	1.30	0.17	1.00	0.00	280.00	8.00	469.20	16.04	0.88	0.08	0.63	0.08	1.00	0.00	
S17	323.20	8.79	612.00	16.97	1.00	0.00	1.31	0.16	1.00	0.00	316.00	4.24	632.80	32.33	0.99	0.01	0.64	0.08	1.00	0.00	
S18	300.00	5.66	620.00	17.15	1.00	0.00	1.32	0.15	1.00	0.00	300.80	4.82	625.20	28.93	1.00	0.00	0.64	0.07	1.00	0.00	
S19	258.80	9.65	473.60	21.51	0.98	0.01	1.29	0.15	1.00	0.00	258.80	13.01	477.20	10.83	0.96	0.02	0.64	0.07	1.00	0.00	
S20	290.00	4.69	562.80	23.35	0.99	0.01	1.28	0.16	1.00	0.00	286.40	2.97	588.00	21.91	0.98	0.02	0.64	0.07	1.00	0.00	
**Mean**	**283.50**	**7.72**	**537.80**	**19.01**	**0.98**	**0.02**	**1.31**	**0.16**	**1.00**	**0.00**	**285.20**	**7.22**	**536.44**	**21.11**	**0.97**	**0.02**	**0.64**	**0.08**	**1.00**	**0.00**	
**SD**	**21.38**	**3.16**	**60.84**	**9.43**	**0.03**	**0.02**	**0.02**	**0.01**	**0.00**	**0.00**	**19.37**	**3.55**	**60.81**	**8.73**	**0.03**	**0.02**	**0.01**	**0.01**	**0.00**	**0.00**	

Specifically, Table 2 shows that the average temporal properties of the N2 component ranged from 163.9 ms to 275.15 ms in ‘Cond1’ and from 165.16 ms to 276.63 ms in ‘Cond2’. Similarly, the average time window for the P3 component (refer to Table 3) ranged from 283.50 ms to 537.80 ms in ‘Cond1’ and from 285.20 ms to 536.44 ms in ‘Cond2’ across all subjects. Evaluation of the inner similarity, an important criterion, across identified time windows for individual subjects showed high reproducibility and consistency in N2 and P3 components. The average inner similarity among subjects was 0.91 and 0.92 for N2 in ‘Cond1’ and ‘Cond2’, respectively. A higher inner similarity was observed for the P3 component, with values of 0.98 and 0.97 for ‘Cond1’ and ‘Cond2’, respectively.

#### Clustering Results for the Real Data

**Fig. 4:**
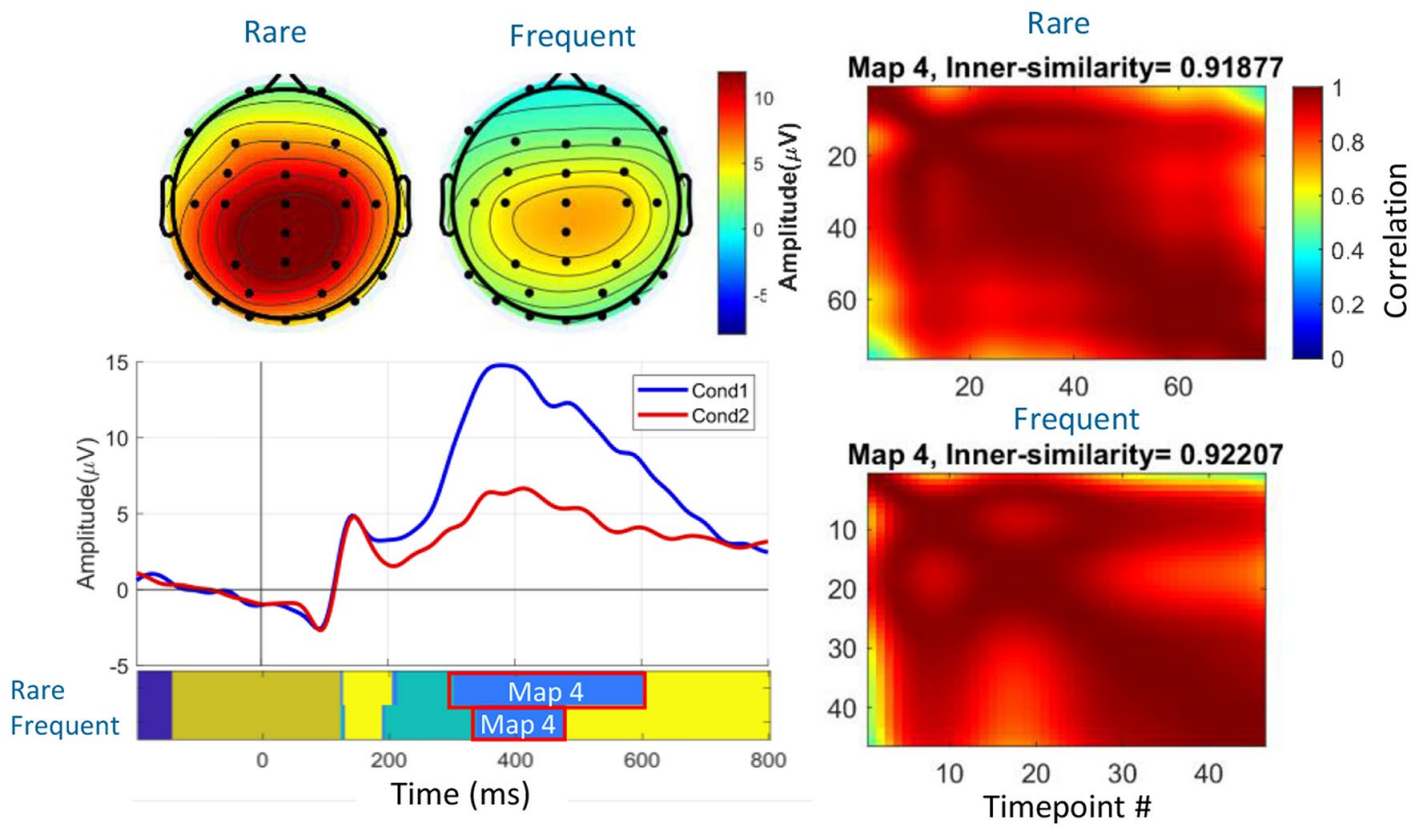
Consensus clustering results on group-averaged ERP data and the identified P3 component derived from the group mean data in six clusters (the optimal number of clusters). The waveform is shown in the Pz electrode. The spatial property of the elicited P3 serves as the template map reference, facilitating the selection of trials and comparison of scoring results (i.e., spatial correlation scores) across individual subjects

Four clustering methods were selected using the M-N plot method for the real data: *k*-means, self-organizing map (SOM), modified *k*-means (with polarity adjustment), and *k*-medoids clustering (KMD). Figure 4 shows the clustering results, determined time windows, topographical maps of the identified P3 component, and the ERP waveform at the Pz electrode. In Fig. 4, cluster maps 4 represent the P3 component for both ‘Rare’ and ‘Frequent’ conditions, with a high inner similarity of 0.92. These identified P3 component properties were used as a reference to analyze the spatial properties of single trials and spatial correlation scores.

**Fig. 5:**
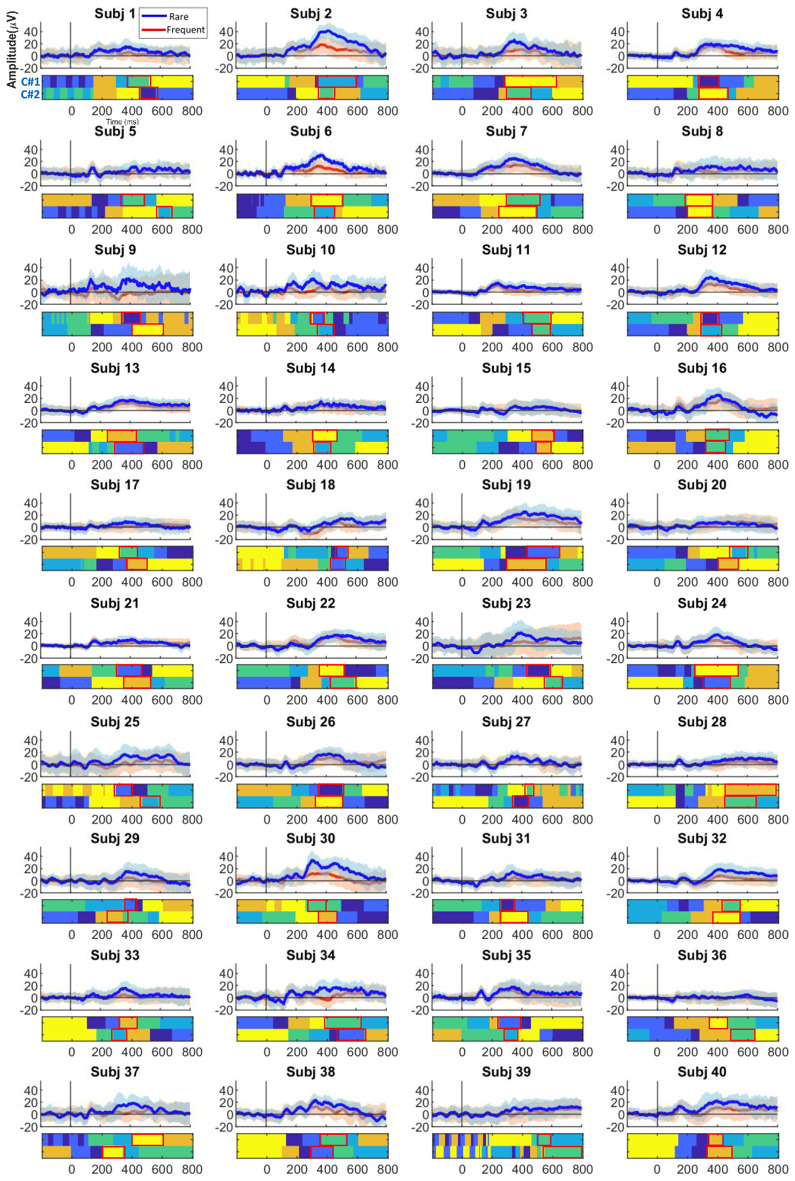
Clustering results in six clusters and estimated time windows (red rectangle) for each subject’s P3 components by condition. ERP and trial waveforms are displayed at the Pz electrode site

At the single-trial clustering level, Table 4 lists the selected clustering methods for each subject’s single trials determined from the M-N plot examination. In cases where no suitable clustering methods were found (e.g., subjects 13, 38, 40), we replaced the clustering list with the methods selected at the group average level. Figure 5 shows the clustering results for individual subjects, presenting ERP waveforms at the Pz electrode with estimated time windows highlighted in red rectangles. The determination of time windows reveals both variety and consistency in the identified ERPs between conditions and across subjects. However, some subjects, such as subject 39, did not exhibit distinct P3 components, possibly due to the absence of highly correlated cluster maps and many noisy clusters. This case will be discussed more in Sect. “Discussion”.

**Table 4:** Clustering methods selected for individual subjects’ ERP data, identified using the M-N plot test in real data. The optimal number of clusters was determined to be six clusters

Subj_ID	Selected methods	Replacement List
S1	KM, SOM, DSPC, SPC, KMD, GMM	-
S2	KM, SOM, DSPC, MKM, KMD, GMM	-
S3	KM, HC, SOM, DSPC, SPC, GMM	-
S4	KM, HC, SOM, DSPC, MKM, GMM	-
S5	SOM, DSPC, MKM, SPC	-
S6	KM, HC, SOM, DSPC, MKM, SPC, KMD, GMM	-
S7	KM, HC, DSPC, MKM, SPC, GMM	-
S8	KM, SOM, DSPC, MKM, SPC, KMD	-
S9	KM, SOM, MKM, KMD, GMM	-
S10	KM, HC, DSPC, MKM, KMD, GMM	-
S11	KM, HC, SOM, MKM, SPC, KMD, GMM	-
S12	DSPC, MKM, SPC, KMD, GMM	-
S13	No Method determined	KM, SOM, MKM, KMD
S14	DSPC, MKM, SPC, KMD, GMM	-
S15	KM, HC, SOM, DSPC, MKM, SPC, KMD, GMM	-
S16	HC, SOM, DSPC, MKM, SPC, KMD, GMM	-
S17	KM, SOM, DSPC, MKM, KMD, GMM	-
S18	KM, SOM, MKM, SPC, KMD	-
S19	DSPC, GMM	-
S20	KM, HC, SOM, DSPC, MKM, KMD, GMM	-
S21	KM, MKM, SPC, KMD, GMM	-
S22	KM, SOM, DSPC, MKM, SPC, KMD, GMM	-
S23	SOM, MKM, KMD	-
S24	KM, HC, SOM, DSPC, MKM, KMD, GMM	-
S25	KM, HC, SOM, DSPC, MKM, KMD, GMM	-
S26	KM, SOM, DSPC, MKM, KMD, GMM	-
S27	KM, HC, SOM, DSPC, GMM	-
S28	KM, HC, SOM, DSPC, MKM, GMM	-
S29	KM, SOM, DSPC, MKM, KMD,GMM	-
S30	KM, HC, SOM, DSPC, MKM, KMD, GMM	-
S31	KM, SOM, DSPC, MKM, KMD, GMM	-
S32	KM, SOM, DSPC, MKM, KMD, GMM	-
S33	HC, SOM, DSPC, KMD, GMM	-
S34	KM, HC, DSPC, SPC GMM	-
S35	KM, HC, SOM, DSPC, MKM, KMD, GMM	-
S36	KM, HC, SOM, MKM, KMD, GMM	-
S37	KM, HC, MKM, SPC, KMD	-
S38	One method (GMM)	KM, SOM, MKM, KMD
S39	KM, HC, SOM, MKM, KMD, GMM	-
S40	One method (DSPC)	KM, SOM, MKM, KMD

Table 5 provides the scoring results, including estimated time windows, the inner similarity of time windows, mean amplitude at the Pz electrode, and spatial correlation between the mean map and the template map topographies, facilitating the qualification of the P3 component for individual subjects. Specifically, the average time window of the P3 components across subjects in the ‘Rare’ condition ranged from 351.08 ms to 495.91 ms, while in the ‘Frequent’ condition, it ranged from 366.01 ms to 498.62 ms. These results indicate suitable consistency across subjects and complement the findings from the group-averaged ERP data in the original study. Additionally, in assessing the quality of the identified cluster maps as the time windows (refer to Table 5), representatives revealed a high inner similarity of the identified time window for the P3 component for the majority of the subjects, averaging 0.91 and 0.92 for the ‘Rare’ and ‘Frequent’ conditions, respectively.

**Table 5:** Scores derived from individual subjects’ determined time windows using the proposed pipeline in real data, encompassing time window properties (start and end), inner similarity, amplitude at Pz electrode, and correlation of mean topography with the template maps

	Rare					Frequent				
Subj-ID	TW start(ms)	TW end(ms)	Innsim	Amp(µv)	Corr	TW start(ms)	TW end(ms)	Innsim	Amp(µv)	Corr
S1	382.03	507.03	0.92	11.12	0.71	456.25	550.00	0.93	2.04	0.53
S2	342.97	577.34	0.98	33.37	0.97	350.78	432.81	0.98	16.50	0.95
S3	288.28	612.50	0.87	15.78	0.91	303.91	444.53	0.87	8.86	0.71
S4	280.47	397.66	0.96	17.79	0.89	280.47	452.34	0.91	13.48	0.82
S5	342.97	467.97	0.86	6.42	0.63	573.44	647.66	0.95	5.82	0.38
S6	303.91	487.50	0.89	23.23	0.84	319.53	444.53	0.92	9.98	0.88
S7	296.09	503.13	0.97	20.29	0.52	249.22	479.69	0.92	12.47	0.65
S8	198.44	346.88	0.83	7.52	0.69	202.34	350.78	0.88	4.12	0.34
S9	342.97	444.53	0.84	18.48	0.47	405.47	604.69	0.90	-1.73	-0.12
S10	303.91	366.41	0.86	17.06	0.73	346.88	428.91	0.92	6.20	0.75
S11	421.09	577.34	0.89	6.68	0.74	471.88	573.44	0.89	3.56	0.50
S12	300.00	389.84	0.97	21.60	0.76	296.09	413.28	0.96	11.66	0.89
S13	241.41	417.19	0.93	13.27	0.78	292.19	464.06	0.94	10.89	0.85
S14	311.72	452.34	0.85	9.39	0.64	319.53	409.38	0.93	6.94	0.85
S15	467.97	596.88	0.87	5.56	0.82	499.22	577.34	0.95	4.71	0.89
S16	327.34	471.88	0.95	20.70	0.74	237.50	319.53	0.91	8.96	0.72
S17	323.44	428.91	0.96	7.35	0.78	374.22	491.41	0.90	1.42	0.56
S18	467.97	526.56	0.90	12.58	0.63	428.91	503.13	0.89	6.24	0.51
S19	436.72	635.94	0.96	20.22	0.91	300.00	546.09	0.93	13.88	0.96
S20	495.31	581.25	0.85	5.36	0.73	409.38	522.66	0.88	7.52	0.88
S21	303.91	452.34	0.89	8.37	0.80	346.88	510.94	0.89	4.61	0.72
S22	350.78	499.22	0.92	14.78	0.92	428.91	573.44	0.96	11.32	0.89
S23	436.72	573.44	0.91	12.24	0.68	553.91	647.66	0.96	9.18	0.28
S24	257.03	522.66	0.86	11.63	0.89	315.63	471.88	0.93	7.39	0.78
S25	292.19	385.94	0.92	11.60	0.66	467.97	565.63	0.88	6.63	0.76
S26	346.88	495.31	0.91	15.60	0.81	319.53	491.41	0.94	9.43	0.64
S27	467.97	514.84	0.84	7.19	0.62	346.88	428.91	0.90	8.98	0.95
S28	452.34	772.66	0.90	8.83	0.49	452.34	643.75	0.93	4.60	0.44
S29	362.50	413.28	0.99	14.10	0.77	339.06	499.22	0.87	2.47	0.56
S30	257.03	479.69	0.92	25.06	0.81	350.78	452.34	0.94	10.91	0.80
S31	260.94	335.16	0.94	7.93	0.77	264.84	428.91	0.95	3.35	0.61
S32	440.63	542.19	0.98	15.44	0.77	378.13	534.38	0.98	6.36	0.66
S33	323.44	425.00	0.93	12.39	0.75	276.56	350.78	0.89	2.59	0.58
S34	389.84	620.31	0.96	13.84	0.77	479.69	639.84	0.87	6.72	0.77
S35	245.31	385.94	0.85	14.49	0.77	288.28	358.59	0.90	11.52	0.79
S36	350.78	460.16	0.89	0.14	0.32	491.41	635.94	0.97	0.05	-0.38
S37	405.47	589.06	0.95	11.75	0.80	210.16	331.25	0.91	1.65	0.27
S38	362.50	510.94	0.92	16.37	0.77	296.09	428.91	0.83	10.40	0.84
S39	510.94	573.44	0.88	8.70	0.82	550.00	796.09	0.86	7.72	0.61
S40	346.88	421.09	0.98	19.80	0.85	335.16	483.59	0.93	9.48	0.91
**Mean**	**351.08**	**495.91**	**0.91**	**13.44**	**0.74**	**366.01**	**498.62**	**0.92**	**7.16**	**0.64**
$\:\widehat{\varvec{S}\varvec{D}}$	**76.12**	**90.64**	**0.05**	**6.34**	**0.13**	**94.32**	**100.99**	**0.03**	**4.03**	**0.28**

### Spatial Properties of Individual Subjects’ ERPs

#### Spatial Properties of ERPs in Simulated Data

**Fig. 6:**
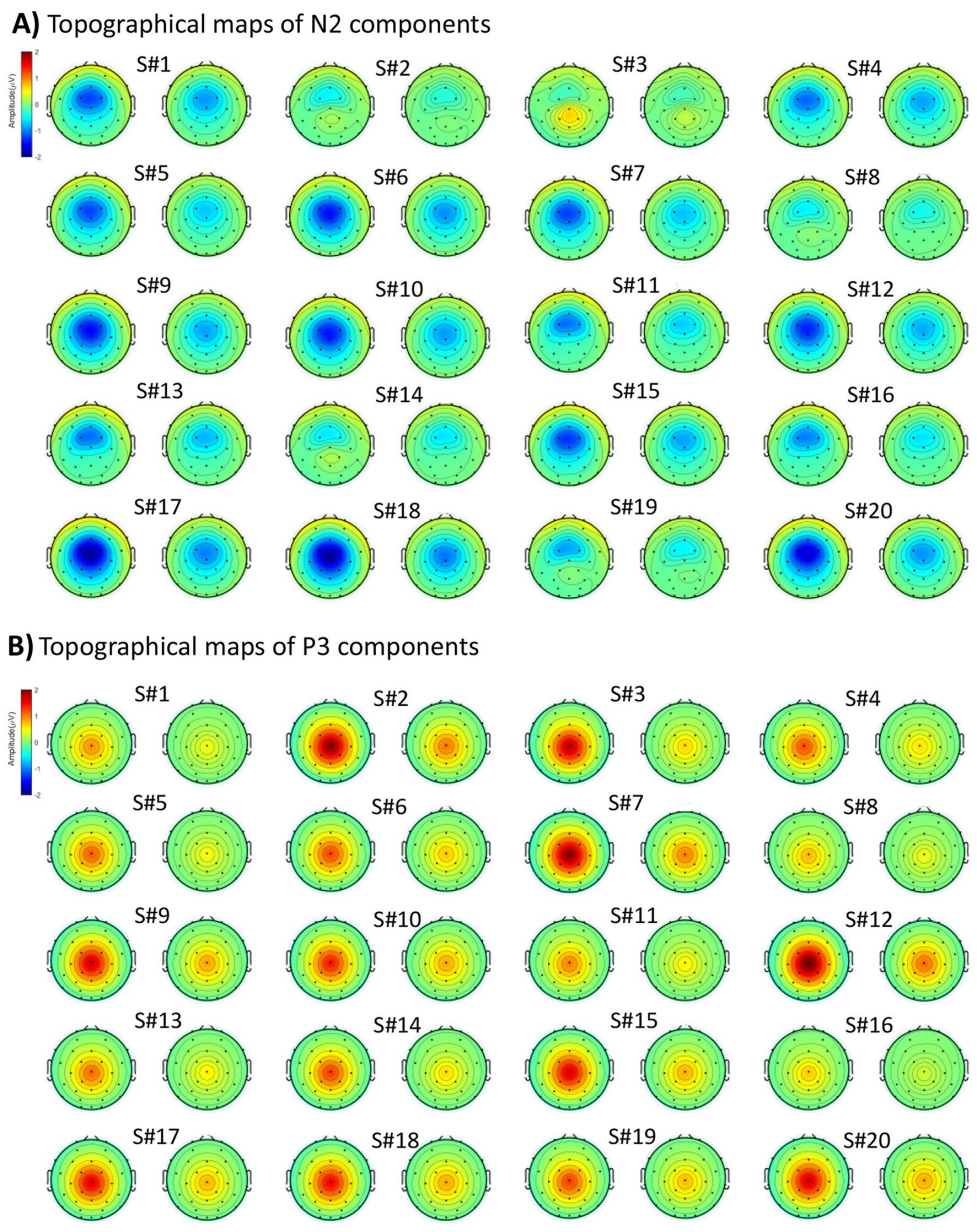
Topographical representation of the ERP components isolated from simulated data (original subjects) under two conditions, highlighting the N2 component (**A**) and the P3 component (**B**). Notably, the topography of both N2 and P3 components is more pronounced in the first condition compared to the second

Figure 6 presents the mean topographical map patterns within the estimated time windows for the individual subjects, illustrating the electrical configuration of the ERP components across subjects. The results shown in Fig. 6 and the spatial correlation scores in Tables 2 and 3 (based on obtained results, e.g., 100 runs of clustering) reveal a high spatial correlation between the topographical maps of most subjects and the pre-defined components (N2 and P3) in the simulated data, particularly with P3 showing a higher correlation than N2 across most subjects.

Specifically, for the identified N2 components from the subjects, the results (see Table 2) revealed a mean correlation of 0.97 and 0.98 for the individual subjects and the corresponding template maps of N2 in ‘Cond1’ and ‘Cond2’, respectively. Notably, a larger negative amplitude was identified in ‘Cond1’ (average − 0.70 µV) compared to ‘Cond2’, with an average of -0.45 µV across most subjects, aligning with the design of the simulated data. Similarly, for identifying the P3 component (refer to Table 3), the results revealed a perfect spatial correlation, with a mean correlation (across subjects) of 1.00 between the individual subjects’ P3 and the corresponding template maps of P3 in both conditions. Additionally, a larger positive amplitude was identified in ‘Cond1’ (average 1.31 µV) compared to ‘Cond2’, with 0.64 µV from the subjects.

#### Spatial Properties of ERP in Real Data

**Fig. 7:**
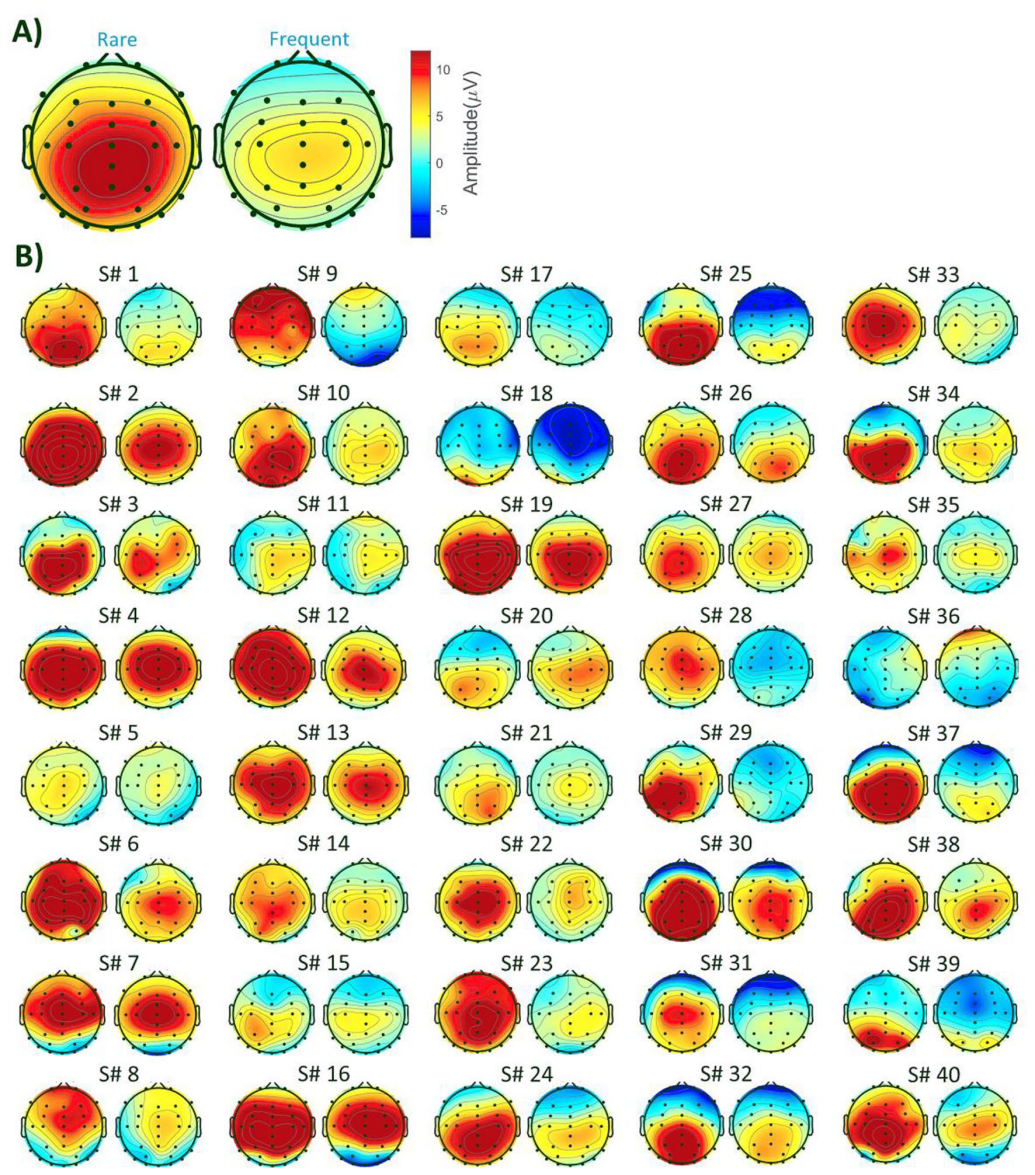
Topographical maps of P3 (within determined time windows) derived from subjects’ ERP data. **A**) Obtained template maps from grand mean ERP data. **B**) Identified P3 topographical maps from individual subjects

For the real data, Fig. 7 and Table 5 illustrate a significant correlation between the individual subjects’ topographical activity and the template maps (topographical maps derived from the group average ERP results). The spatial analysis conducted on the identified P3 components of individual subjects revealed a reasonable correlation between the subjects and the template maps, averaging 0.74 in the ‘Rare’ condition and 0.64 in the ‘Frequent’ condition. Notably, the correlation between the topography of P3 and the template map was not observed in some subjects, such as subjects 9 and 36, suggesting potential overlapping components or diminished brain responses in the trials. We will discuss this in Sect. “Discussion” in more detail. Additionally, a larger amplitude was observed in the ‘Rare’ condition (average 13.44 µV) compared to the amplitude in the ‘Frequent’ condition (average 7.16 µV) across most subjects, indicating a consistent effect size in the majority of the subjects in the determined time windows.

### Evaluation Metrics and Performance Results

Here, we present the performance results, including the scoring and statistical analysis outcomes for the simulated data, along with analytical tests and statistical tests for the real data. First, we provide the designed Monte Carlo test results for the simulated data, followed by the performance results.

#### Performance Results for the Simulated and Real Data

**Fig. 8:**
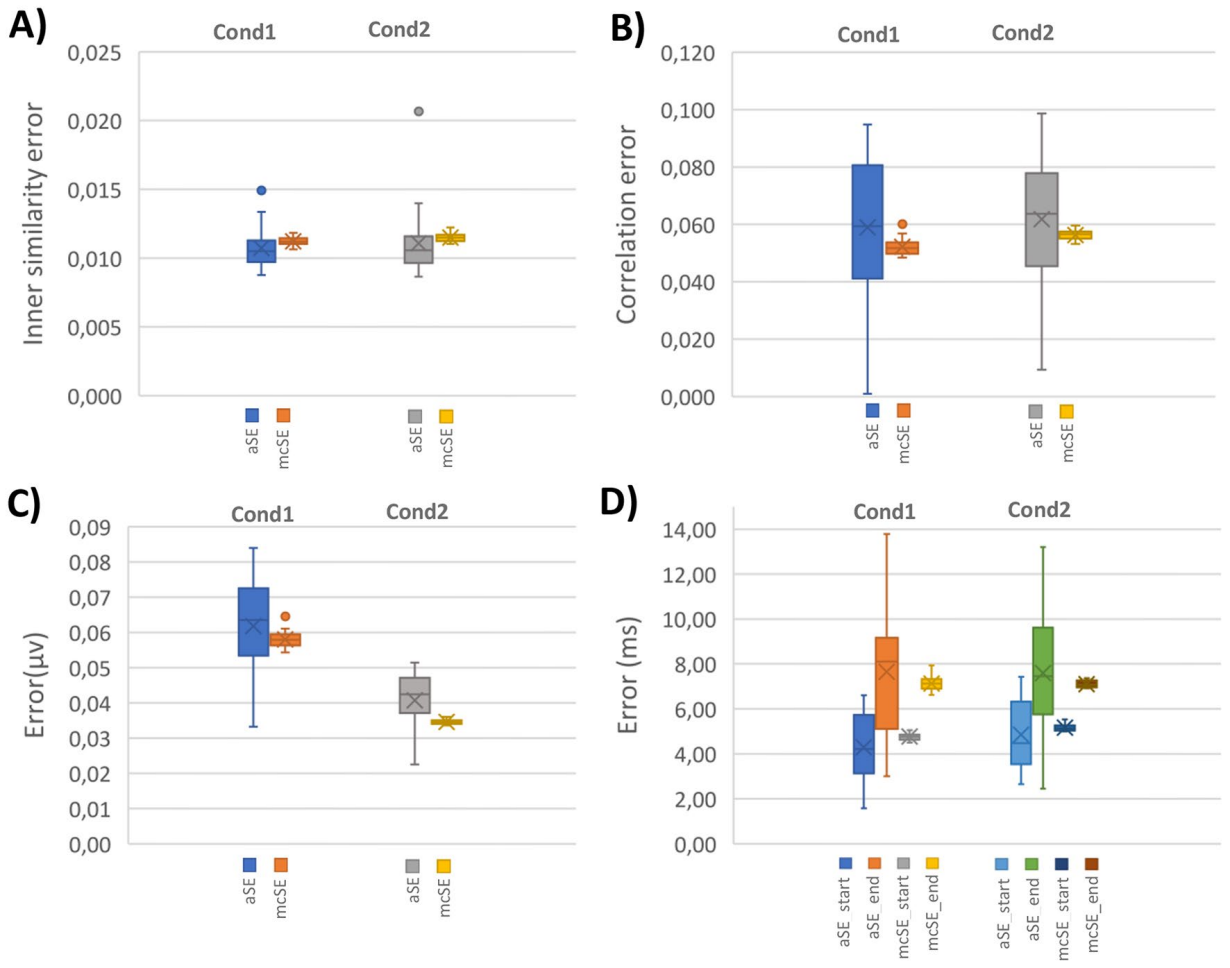
Comparison of analytical standard measurement error ($\:\widehat{aSE}$) and Monte Carlo SE ($\:\widehat{mcSE}$) for N2 component scores in simulated data. **A)**$\:\widehat{SEs}$ for inner similarity scores from single trials’ estimated time windows in 1000 Monte Carlo iterations. **B)**$\:\widehat{SEs}$ for spatial correlation scores with pre-defined N2 from estimated time windows. **C)**$\:\widehat{SEs}$ for amplitude scores at Cz electrode site from mean topography within the estimated time window. **D)**$\:\widehat{SEs}$ for latency scores at the ‘start’ and ‘end’ of the estimated time window

**Fig. 9:**
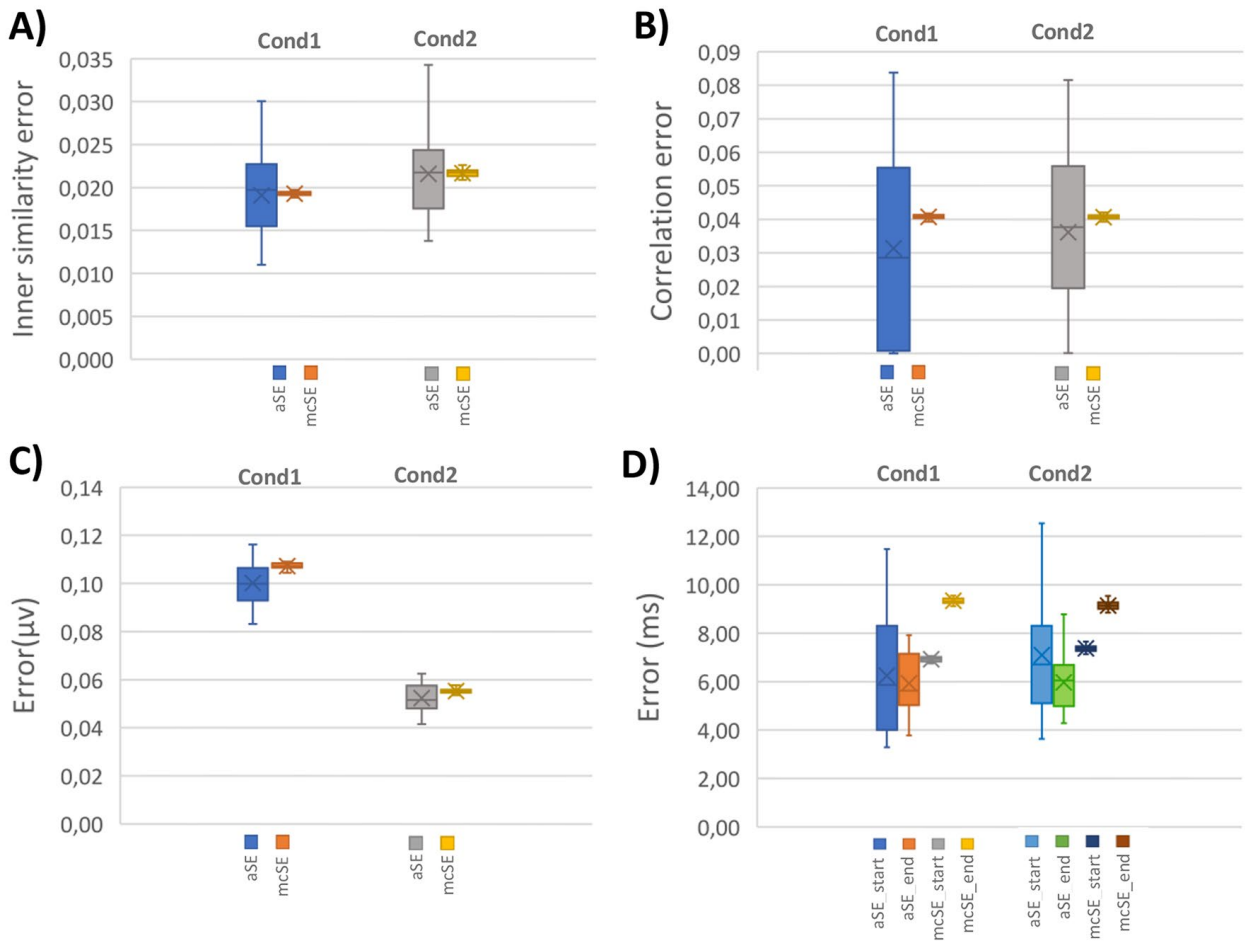
Comparison of analytical standard measurement error ($\:\widehat{aSE}$) and Monte Carlo SE ($\:\widehat{mcSE}$) for P3 component scores in simulated data. **A**) $\:\widehat{SEs}$ for inner similarity scores from single trials’ estimated time windows in 1000 Monte Carlo iterations. **B**) $\:\widehat{SEs}$ for spatial correlation scores with pre-defined P3 from estimated time windows. **C**) $\:\widehat{SEs}$ for amplitude scores at Pz electrode site from mean topography within the estimated time window. **D**) $\:\widehat{SEs}$ for latency scores at the ‘start’ and ‘end’ of the estimated time window

Figures 8 and 9 compare the scoring items, including analytical scores derived from subjects’ trials and Monte Carlo scores obtained through 1000 iterations of trial clustering *with replacement*. The scoring items encompassed mean amplitude, inner similarity, time window properties, and correlation (i.e., between the mean topography of identified N2 and P3 and pre-defined components). The $\:aSE$ results were derived from single trials of individual subjects, while $\:mcSEs$ were obtained from the Monte Carlo procedure. Our aim in evaluating the $\:SE$ of the scores is to understand how the scores might fluctuate with repeated experiments (in terms of processing method). Additionally, the repeated measurement offers an overall estimation of the scores through the Monte Carlo test, signifying the consistency in scoring results from single-trial cluster analysis. From the experimental design perspective, this can indicate the quality of experiment conduction and signal processing performance.

To assess the difference between the obtained corresponding $\:\widehat{aSE}$ and $\:\widehat{mcSE}$, we conducted *two*-sample *t*-tests. The statistical test revealed no significant difference between the obtained scores from analytical and Monte Carlo testing results for N2 identification. However, for the P3 component, a significant difference (*p*-value = 0.000) was observed in the time window endpoint property due to variation in the identified endpoints of P3 from both conditions. Furthermore, a significant difference (*p*-value < 0.001) was noted between the calculated amplitudes using analytical and Monte Carlo test methods in ‘Cond1’. Additional details can be found in the supplementary Tables S1 to S8.

Further reliability tests on the obtained scores from different items in Tables 2 and 3 revealed a Cronbach’s alpha of 0.59 for N2 and 0.74 for P3. These values were derived from the scores in two conditions, indicating relatively consistent results in P3 compared to N2, although not necessarily high, across subjects. This variability between subjects was pre-defined based on the nature of the subjects’ evoked responses. A similar interpretation is valid, as Cronbach’s alpha, calculated from the real data for the obtained scores (Table 5), was 0.70.

#### Statistical Analysis Results in Simulated and Real Data

Two sets of repeated measures ANOVA were conducted on the simulated data. First, statistical analysis was performed using analytical results, and second, the ANOVA test was applied to timing scores from the Monte Carlo test. The mean amplitude within the selected time windows and the electrode sites from individual subjects was calculated for the statistical test.

For the simulated data, the results from the analytical latencies of the subjects revealed a significant main effect of condition for N2 (F(1,19) = 22.26, *p*-value < 0.0001, $\:{\eta\:}_{p}^{2}$= 0.463) and for P3 (F(1,19)= 219.40, *p*-value = 0.00, $\:{\eta\:}_{p}^{2}$= 0.914). Similarly, significant main effects of condition for N2 (F(1,19)= 64.43, *p*-value < 0.0001, $\:{\eta\:}_{p}^{2}$= 0.742) and P3 (F(1,19)= 115.26, *p*-value = 0.00, $\:{\eta\:}_{p}^{2}$= 0.853) were found from the Monte Carlo scores. As expected from the simulation design, a larger potential was elicited in the first condition for both N2 and P3 components. Note that we have reported the averaged results from 1000 iterations for both sets of statistical analyses (simulated subjects and Monte Carlo-generated subjects) independently.

For the real data, the repeated measures ANOVA on the selected time windows from individual subjects revealed a significant main effect of the stimulus (F(1,39) = 74.69, *p*-value < 0.0001, $\:{\eta\:}_{p}^{2}$= 0.651), indicating a large effect of the P3 component. Notably, a large positive potential in the central lobe region was observed in the ‘Rare’ condition compared to the ‘Frequent’ condition, confirming previous findings from the original study.

Hence, the statistical analysis results from both simulated and real data underscore the recognition of individual variability in the precise timing of neural processes associated with given specific cognitive functions using single-trial cluster analysis.

## Discussion

We introduced a multi-set consensus clustering pipeline for analyzing single-trial EEG data to quantify brain-evoked responses in individual subjects. Our approach involved applying consensus clustering first at the single-trial level and then combining these results across trials through subject-level consensus clustering. This method aims to extract consistent cognitive responses by identifying the consecutive time points with stable contributions across trials, aggregating clustering outcomes, and mitigating the impact of noisy clusters. We evaluated our method using simulated and real data to quantify ERP components and conduct reproducibility tests. Through Monte Carlo and analytical tests, we demonstrated the consistency and robustness of our pipeline, providing reliable clustering and scoring results from evoked single-trial EEG epochs of individuals. Notably, the estimated time windows offered a realistic representation of individual subjects’ brain activities, making them suitable for both group-level and individual analyses rather than relying on constant measurement intervals for all subjects.

The proposed method differs from conventional approaches in two main aspects. Firstly, it explores the spatial and temporal properties of cognitive processes from single-trial EEG data at the individual subject level. This is achieved by investigating mutual temporal information from single trials and assessing inner similarity (stable spatial configuration) during time window determination. In contrast, conventional microstate analysis methods typically focus on evaluating spatial properties to classify microstates into dominant classes of maps (e.g., four classic classes) for event-related and resting-state EEG (Antonova et al. 2022; Michel and Koenig 2018; Zappasodi et al. 2019). Specifically, for ERP data, the microstate analysis method assigns GFP points from individual subjects’ ERP data into template maps obtained from clustering group average ERP data (Murray et al., 2008; Ruggeri et al. 2019). Thus, the temporal structure of the ERP of interest is statistically identified where specific topography is dominant, typically obtained through clustering of single-trial data. Meanwhile, identifying the temporal occurrence of template maps relies on statistical analysis (De Lucia et al. 2007a; Tzovara et al., 2012a; Tzovara et al. 2012b).

Secondly, the proposed pipeline incorporates an adaptive clustering configuration during the consensus clustering generation phase for each subject. This involves using the M-N plot-based clustering selection and a data-driven approach to determine the optimal number of clusters (Mahini et al. 2022b). In contrast, conventional microstate studies and consensus clustering methods on EEG/ERP data typically adopt a fixed set of clustering methods for all subjects (Koenig et al. 2014; Mahini et al. 2022b; Ruggeri et al. 2019). It is worth noting that the proposed method may encounter suboptimal clustering performance in low SNR data, leading to many noisy clusters—a common challenge in clustering-based approaches (Mahini et al. 2023). To mitigate this, we introduced a post-hoc processing step that can be applied at different clustering levels. This step involves identifying thin cluster maps with a small number of samples (e.g., < 10 ms) and assigning them to neighboring cluster maps if they exhibit sufficiently high spatial correlation (e.g., > 0.90 between mean topography maps).

Another consideration involves the challenge of identifying highly overlapped components using clustering methods, given the variability in individual subjects. This challenge arises because real brain responses can be mixed with other components, particularly during the processing of group average ERP data where trials from all subjects are averaged. Our approach addresses this by analyzing individual subjects’ responses from actual trials, thus recognizing the diverse timing of neural processes across individuals and providing a more precise representation of their cognitive functions compared to traditional averaging methods.

The reproducibility test results from both simulated and real data demonstrated the robustness of the proposed pipeline, as evidenced by stable analytical and Monte Carlo scores (refer to supplementary materials, Table S1 to S8). The variability observed across subjects, particularly in spatial correlation, aligned with experimental expectations. However, anomalies were noted in a few subjects where the corresponding Monte Carlo standard error $\:\widehat{mcSE}$ did not necessarily indicate lower values than the analytical standard error $\:\widehat{aSE}$. For instance, subjects 9 and 39 displayed relatively aberrant results in the real data. These divergences could arise from two potential factors. Firstly, the obtained topographical maps might have exhibited low$\:SNR$ and lacked statistical reliability within the estimated time windows. Secondly, the trials selected during the preprocessing phase may not have contained sufficiently strong ERP responses, potentially leading to the inclusion of trials with lower spatial correlation to maintain a minimum number of individual trials. Lower $\:\widehat{mcSEs}$ were interpreted as indicative of greater reproducibility in the clustering results of the selected trials and the obtained scores.

Moreover, the determination of significant effect sizes for N2 and P3 in simulated data, coupled with Monte Carlo testing, demonstrated suitable stability across all examined score items obtained from single-trial EEG epochs. Monte Carlo testing in simulated data reaffirmed the reliability of quantifying N2 and P3 across both conditions while using multi-set consensus clustering. The developed pipeline elucidated spatially correlated brain activity with similar temporal properties (though not necessarily identical), supporting the idea of consistent brain responses across single trials and individual subjects. The reproducibility assessment highlighted result consistency, indicating the reliability of the proposed cluster analysis with an iterative generation of random trials.

From a statistical analysis perspective, two sets of statistical analyses from the estimated time windows of the subjects (i.e., from real simulated and randomized generating trials) disclosed significant main effects of N2 and P3 (see Table 6) in the simulated data. Importantly, the statistical analysis in the real data highlighted a significant effect alongside the identified time windows, which showed a larger positive potential in the ‘Rare’ condition compared to the ‘Frequent’ condition in most subjects, thereby confirming the findings of a previous study (Kappenman et al. 2021). Ultimately, our method is not confined to identifying the standard P3 component, as demonstrated in this study; it holds the potential for identifying other ERP components from event-related single-trial EEG data. Furthermore, the proposed method instills confidence in exploring the ERP of interest for individual subjects, which is crucial for various individual subject investigations. However, more comprehensive studies and reliability tests are warranted to address potential risks and ethical concerns before deploying this method in critical applications.

**Table 6:** Statistical analysis results from repeated measures ANOVA tests on the estimated time windows and electrode sites (Fz for N2 and cz for P3 components) of individual subjects comparing Monte Carlo simulations with actual subject data in the simulated data (number of iterations = 1000)

ERP	*P*-value (SD)	F-value (SD)	Eta2(SD)
N2	**0.001** (0.00)	22.26 (20.55)	0.463 (0.20)
P3	**0.000** (0.00)	219.40 (63.10)	0.914 (0.02)
ANOVA results from Monte-Carlo the generated subjects			
N2	**0.001** (0.00)	64.43 (26.14)	0.742 (0.11)
P3	**0.000** (0.00)	115.26 (27.02)	0.853 (0.03)

## Conclusions

Our method successfully addresses the challenge of identifying ERPs of interest from single-trial EEG data by integrating clusterings investigated from individual trials, even with minimal prior knowledge about the component of interest. Our findings suggest that single-trial EEG clustering can reliably identify evoked responses in individual subjects. The results affirm the presence of spatially correlated cluster maps in single trials of individual subject data, indicating appropriate estimations of brain responses. Furthermore, our pipeline enhances the likelihood of detecting the real components by providing an unbiased approach to identifying interesting ERPs. This study holds promise as a valuable tool for reliably investigating individual subject brain activity, particularly in clinical applications, which remain open research questions in single-trial EEG data analysis. Future advancements may take advantage of multi-dimensional single-trial EEG processing, offering a robust method to explore brain responses across various domains and perspectives through clustering analyses.

## Electronic Supplementary Material

Below is the link to the electronic supplementary material.

Supplementary Material 1
