# Supplementary material for: Brain Evoked Response Qualification Using Multi-Set Consensus Clustering: Toward Single-Trial EEG Analysis
Source: Brain Topogr. 2024 Aug 20;37(6):1010–32. doi: 10.1007/s10548-024-01074-y (PMC11408575; doi:10.1007/s10548-024-01074-y)
Supplement: Supplementary file 1 — Supplementary Material 1 [file 10548_2024_1074_MOESM1_ESM.docx]

**Supplementary Materials**

**Tables captions**

**Table S1.** Latency scores and measurement errors (i.e., including $\hat{aSE}$ as analytical and $\hat{mcSE}$ the Monte Carlo test error) for the identified N2 in individual subjects. The $\hat{aSE}$ is obtained from processing all the trials, and the $\hat{mcSE}$ is calculated from the Monte Carlo. In addition, the root mean square error (RMS) is calculated from the scores $SE$. Cond1=condition 1, Cond2=condition 2, TW= time window, $\hat{aSE}$= estimated analytical standard error, $\hat{mcSE}$= estimated standard error from Monte Carlo, Subj_ID= subject ID, SD= standard deviation, RMS= root mean square.

**Table S2.** Illustration of the latency scores and the calculated measurement errors (i.e., including $\hat{aSE}$ as analytical and $\hat{mcSE}$ the Monte Carlo test error) for the identified P3 in individual subjects.

**Table S3.** Calculated $\hat{aSE}$ and $\hat{mcSE}$ from the obtained mean amplitude of N2 in Fz electrode site in the estimated time windows of the individual subjects.

**Table S4.** Calculated $\hat{aSE}$ and $\hat{mcSE}$ from the obtained mean amplitude of P3 in Cz electrode site in the estimated time windows of the individual subjects.

**Table S5.** The obtained $\hat{aSE}$ and $\hat{mcSE}$ of spatial correlation of N2, i.e., the correlation between the estimated N2 and ground truth topography (predefined N2) in the simulated data.

**Table S6.** The obtained $\hat{aSE}$ and $\hat{mcSE}$ of spatial correlation between the estimated and predefined P3 component in the simulated data.

**Table S7.** Calculated $\hat{aSE}$ and $\hat{mcSE}$ results from the inner similarity of the N2 component in the estimated time window of the individual subjects.

**Table S8.** The obtained $\hat{aSE}$ and $\hat{mcSE}$ results from the inner similarity scores of the P3 component in the estimated time window of the individual subjects.

**Table S1.** Latency scores and measurement errors (i.e., including $\hat{aSE}$ as analytical and $\hat{mcSE}$ the Monte Carlo test error) for the identified N2 in individual subjects. The $\hat{aSE}$ is obtained from processing all the trials, and the $\hat{mcSE}$ is calculated from the Monte Carlo. In addition, the root mean square error (RMS) is calculated from the scores $SE$. Cond1=condition 1, Cond2=condition 2, TW= time window, $\hat{aSE}$= estimated analytical standard error, $\hat{mcSE}$= estimated standard error from Monte Carlo, Subj_ID= subject ID, SD= standard deviation, RMS= root mean square.

|  | **Cond1** | | | | | | **Cond2** | | | | | |
| --- | --- | --- | --- | --- | --- | --- | --- | --- | --- | --- | --- | --- |
| **Subj_ID** | **TW**  **(start/ms)** | **TW**  **(end/ms)** | $\hat{\boldsymbol{aSE}}$  **start** | $\hat{\boldsymbol{aSE}}$  **end** | $\hat{\boldsymbol{mcSE}}$  **start** | $\hat{\boldsymbol{mcSE}}$  **end** | **TW**  **(start/ms)** | **TW**  **(end/ms)** | $\hat{\boldsymbol{aSE}}$  **start** | $\hat{\boldsymbol{aSE}}$  **end** | $\hat{\boldsymbol{mcSE}}$  **start** | $\hat{\boldsymbol{mcSE}}$  **end** |
| S1 | 163.52 | 270.64 | 2.67 | 8.53 | 4.61 | 7.34 | 162.16 | 276.78 | 3.08 | 7.49 | 5.34 | 7.18 |
| S2 | 164.62 | 275.50 | 6.61 | 13.78 | 5.05 | 7.93 | 164.12 | 277.34 | 7.43 | 13.21 | 5.18 | 6.93 |
| S3 | 162.86 | 273.88 | 6.04 | 10.40 | 4.88 | 7.25 | 161.26 | 278.20 | 6.92 | 10.42 | 5.22 | 6.95 |
| S4 | 162.92 | 272.06 | 3.45 | 11.47 | 4.78 | 6.75 | 162.94 | 276.88 | 3.91 | 9.82 | 5.32 | 6.98 |
| S5 | 164.48 | 276.32 | 3.54 | 8.38 | 4.75 | 7.03 | 163.30 | 278.24 | 3.45 | 7.41 | 5.53 | 7.37 |
| S6 | 163.80 | 276.18 | 4.74 | 8.62 | 4.64 | 7.11 | 163.86 | 277.84 | 5.59 | 9.98 | 5.35 | 7.31 |
| S7 | 162.74 | 272.14 | 3.98 | 4.68 | 4.50 | 6.62 | 162.22 | 277.24 | 4.42 | 6.43 | 5.29 | 7.01 |
| S8 | 164.34 | 273.70 | 5.32 | 8.44 | 4.76 | 6.98 | 162.86 | 275.40 | 6.01 | 9.03 | 5.12 | 6.95 |
| S9 | 163.20 | 271.44 | 5.42 | 5.28 | 4.82 | 7.24 | 162.74 | 277.10 | 5.68 | 6.65 | 5.04 | 6.99 |
| S10 | 162.60 | 272.56 | 5.83 | 7.59 | 4.73 | 7.37 | 162.58 | 278.42 | 6.41 | 8.40 | 5.10 | 7.10 |
| S11 | 163.02 | 272.70 | 1.83 | 7.85 | 4.92 | 7.41 | 163.20 | 277.04 | 2.79 | 7.50 | 5.01 | 7.08 |
| S12 | 163.60 | 272.96 | 6.10 | 5.05 | 4.61 | 7.07 | 162.20 | 277.12 | 6.26 | 4.99 | 5.16 | 7.06 |
| S13 | 165.28 | 273.74 | 6.14 | 6.93 | 4.62 | 6.74 | 163.14 | 277.84 | 6.59 | 5.29 | 5.22 | 7.36 |
| S14 | 163.70 | 276.02 | 4.21 | 9.34 | 4.79 | 6.91 | 163.82 | 276.90 | 4.21 | 8.11 | 5.07 | 7.02 |
| S15 | 162.76 | 271.44 | 4.79 | 9.54 | 4.97 | 7.59 | 162.90 | 274.92 | 6.34 | 10.72 | 5.04 | 6.92 |
| S16 | 164.84 | 271.84 | 2.75 | 6.46 | 4.77 | 6.91 | 162.74 | 276.78 | 2.67 | 5.66 | 5.05 | 7.31 |
| S17 | 163.36 | 271.76 | 4.22 | 4.77 | 4.62 | 6.83 | 163.58 | 275.82 | 4.43 | 5.81 | 5.01 | 7.27 |
| S18 | 163.04 | 272.18 | 3.68 | 3.01 | 4.81 | 7.27 | 162.04 | 276.40 | 4.53 | 2.46 | 5.16 | 6.92 |
| S19 | 165.38 | 272.96 | 3.02 | 8.35 | 4.88 | 7.20 | 162.44 | 276.26 | 3.79 | 6.91 | 5.11 | 7.28 |
| S20 | 162.90 | 271.94 | 1.58 | 4.52 | 4.82 | 7.16 | 162.72 | 277.20 | 2.65 | 5.75 | 5.00 | 7.13 |
| **Mean** | **163.65** | **273.10** | **4.30** | **7.65** | **4.77** | **7.14** | **162.84** | **276.99** | **4.86** | **7.60** | **5.17** | **7.11** |
| **SD** | **0.88** | **1.70** | **1.49** | **2.64** | **0.14** | **0.31** | **0.70** | **0.92** | **1.54** | **2.45** | **0.14** | **0.16** |
| **RMS(**$\hat{\boldsymbol{SE}}$**)** | **-** | - | **4.53** | **8.07** | **4.77** | **7.14** | **-** | - | **5.09** | **7.97** | **5.17** | **7.11** |

**Table S2.** Illustration of the latency scores and the calculated measurement errors (i.e., including $\hat{aSE}$ as analytical and $\hat{mcSE}$ the Monte Carlo test error) for the identified P3 in individual subjects.

|  | **Cond1** | | | | | | | **Cond2** | | | | | |
| --- | --- | --- | --- | --- | --- | --- | --- | --- | --- | --- | --- | --- | --- |
| **Subj_ID** | **TW**  **(start/ms)** | | **TW**  **(end/ms)** | $\hat{\boldsymbol{aSE}}$  **start** | $\hat{\boldsymbol{aSE}}$  **end** | $\hat{\boldsymbol{mcSE}}$  **start** | $\hat{\boldsymbol{mcSE}}$  **end** | **TW**  **(start/ms)** | **TW**  **(end/ms)** | $\hat{\boldsymbol{aSE}}$  **start** | $\hat{\boldsymbol{aSE}}$  **end** | $\hat{\boldsymbol{mcSE}}$  **start** | $\hat{\boldsymbol{mcSE}}$  **end** |
| S1 | 285.15 | | 554.99 | 8.30 | 7.69 | 6.78 | 9.16 | 286.93 | 559.78 | 9.80 | 7.52 | 7.43 | 9.26 |
| S2 | 285.91 | | 554.89 | 5.21 | 5.05 | 6.93 | 9.31 | 286.78 | 561.46 | 5.62 | 4.58 | 7.66 | 9.54 |
| S3 | 285.20 | | 554.89 | 9.61 | 3.78 | 6.82 | 9.30 | 286.22 | 556.22 | 11.52 | 4.36 | 7.41 | 9.15 |
| S4 | 285.20 | | 556.19 | 6.22 | 5.43 | 6.91 | 9.31 | 286.44 | 562.83 | 7.63 | 5.67 | 7.26 | 8.98 |
| S5 | 285.20 | | 554.79 | 6.14 | 7.56 | 7.00 | 9.44 | 287.09 | 558.92 | 7.32 | 6.74 | 7.59 | 9.34 |
| S6 | 285.36 | | 553.45 | 3.75 | 6.68 | 6.95 | 9.41 | 286.19 | 558.92 | 5.55 | 6.29 | 7.18 | 8.97 |
| S7 | 285.22 | | 554.35 | 3.98 | 5.63 | 6.82 | 9.17 | 286.52 | 558.52 | 5.07 | 6.52 | 7.39 | 9.14 |
| S8 | 285.56 | | 554.87 | 11.48 | 4.19 | 6.75 | 9.11 | 287.06 | 560.59 | 12.54 | 4.28 | 7.19 | 8.93 |
| S9 | 285.10 | | 555.11 | 4.09 | 7.26 | 6.81 | 9.22 | 286.85 | 560.59 | 4.61 | 7.71 | 7.59 | 9.41 |
| S10 | 284.94 | | 554.50 | 3.32 | 7.19 | 7.02 | 9.44 | 286.49 | 560.57 | 6.27 | 6.91 | 7.41 | 9.20 |
| S11 | 284.82 | | 555.18 | 9.11 | 5.25 | 6.86 | 9.23 | 287.28 | 558.69 | 7.14 | 4.98 | 7.14 | 8.84 |
| S12 | 285.58 | | 555.46 | 3.29 | 5.62 | 7.01 | 9.41 | 287.08 | 559.86 | 3.63 | 6.19 | 7.26 | 9.05 |
| S13 | 285.68 | | 554.63 | 7.54 | 5.39 | 6.95 | 9.31 | 287.35 | 558.89 | 8.25 | 5.55 | 7.39 | 9.09 |
| S14 | 285.51 | | 554.12 | 8.29 | 5.02 | 6.87 | 9.31 | 286.69 | 557.53 | 8.21 | 5.47 | 7.35 | 9.05 |
| S15 | 285.17 | | 554.56 | 5.59 | 6.44 | 7.06 | 9.49 | 286.62 | 560.55 | 6.29 | 6.10 | 7.24 | 9.00 |
| S16 | 285.41 | | 554.52 | 9.19 | 4.39 | 7.02 | 9.38 | 286.51 | 558.12 | 10.05 | 4.68 | 7.42 | 9.15 |
| S17 | 285.72 | | 555.18 | 4.37 | 7.91 | 7.02 | 9.56 | 286.07 | 558.17 | 4.62 | 8.78 | 7.30 | 9.02 |
| S18 | 284.90 | | 554.27 | 4.63 | 7.00 | 6.86 | 9.31 | 287.19 | 559.15 | 5.19 | 6.13 | 7.46 | 9.28 |
| S19 | 284.86 | | 555.07 | 7.04 | 4.90 | 6.93 | 9.40 | 286.59 | 558.48 | 8.31 | 5.02 | 7.48 | 9.28 |
| S20 | 285.27 | | 553.77 | 3.83 | 6.22 | 7.04 | 9.45 | 285.69 | 558.33 | 4.19 | 5.99 | 7.38 | 9.14 |
| **Mean** | **285.29** | | **554.74** | **6.25** | **5.93** | **6.92** | **9.34** | **286.68** | **559.31** | **7.09** | **5.97** | **7.38** | **9.14** |
| **SD** | **0.30** | | **0.60** | **2.44** | **1.24** | **0.09** | **0.12** | **0.43** | **1.50** | **2.47** | **1.19** | **0.14** | **0.17** |
| **RMS(**$\hat{\boldsymbol{SE}}$**)** | | **-** | **-** | **6.69** | **6.05** | **6.92** | **9.34** | **-** | - | **7.49** | **6.09** | **7.38** | **9.14** |

**Table S3.** Calculated $\hat{aSE}$ and $\hat{mcSE}$ from the obtained mean amplitude of N2 in Fz electrode site in the estimated time windows of the individual subjects.

|  | **Cond1** | | | **Cond2** | | |
| --- | --- | --- | --- | --- | --- | --- |
| **Subj_ID** | **Score (µv)** | $\hat{\mathbf{aSE}}\mathbf{(}\boldsymbol{\mu v}\mathbf{)}$ | $\hat{\mathbf{mcSE(}\boldsymbol{\mu v}\mathbf{)}}$ | **Score( µv)** | $\hat{\mathbf{aSE(}\boldsymbol{\mu v}\mathbf{)}}$ | $\hat{\mathbf{mcSE(}\boldsymbol{\mu v}\mathbf{)}}$ |
| S1 | -0.71 | 0.07 | 0.06 | -0.45 | 0.04 | 0.03 |
| S2 | -0.70 | 0.08 | 0.06 | -0.45 | 0.05 | 0.03 |
| S3 | -0.71 | 0.08 | 0.06 | -0.43 | 0.05 | 0.03 |
| S4 | -0.73 | 0.07 | 0.06 | -0.45 | 0.05 | 0.03 |
| S5 | -0.69 | 0.05 | 0.06 | -0.45 | 0.03 | 0.04 |
| S6 | -0.69 | 0.06 | 0.06 | -0.45 | 0.04 | 0.04 |
| S7 | -0.70 | 0.06 | 0.05 | -0.44 | 0.05 | 0.03 |
| S8 | -0.69 | 0.07 | 0.06 | -0.46 | 0.04 | 0.03 |
| S9 | -0.71 | 0.03 | 0.06 | -0.45 | 0.03 | 0.03 |
| S10 | -0.70 | 0.05 | 0.06 | -0.44 | 0.04 | 0.03 |
| S11 | -0.71 | 0.07 | 0.06 | -0.45 | 0.05 | 0.03 |
| S12 | -0.71 | 0.05 | 0.06 | -0.45 | 0.03 | 0.03 |
| S13 | -0.69 | 0.08 | 0.06 | -0.45 | 0.04 | 0.04 |
| S14 | -0.69 | 0.08 | 0.06 | -0.45 | 0.05 | 0.03 |
| S15 | -0.71 | 0.07 | 0.06 | -0.45 | 0.05 | 0.03 |
| S16 | -0.71 | 0.06 | 0.06 | -0.45 | 0.04 | 0.04 |
| S17 | -0.70 | 0.06 | 0.05 | -0.45 | 0.04 | 0.04 |
| S18 | -0.71 | 0.03 | 0.06 | -0.45 | 0.02 | 0.03 |
| S19 | -0.70 | 0.07 | 0.06 | -0.45 | 0.05 | 0.03 |
| S20 | -0.71 | 0.05 | 0.06 | -0.45 | 0.04 | 0.03 |
| **Mean** | **-0.70** | **0.06** | **0.06** | **-0.45** | **0.04** | **0.03** |
| **SD** | **0.01** | **0.01** | **0.00** | **0.01** | **0.01** | **0.00** |
| **RMS(**$\hat{\boldsymbol{SE}}$**)** | **-** | **0.06** | **0.06** | **-** | **0.04** | **0.03** |

**Table S4.** Calculated $\hat{aSE}$ and $\hat{mcSE}$ from the obtained mean amplitude of P3 in Cz electrode site in the estimated time windows of the individual subjects.

|  | **Cond1** | | | **Cond2** | | |
| --- | --- | --- | --- | --- | --- | --- |
| **Subj_ID** | **Score (µv)** | $\hat{\mathbf{aSE}}\mathbf{(}\boldsymbol{\mu v}\mathbf{)}$ | $\hat{\mathbf{mcSE(}\boldsymbol{\mu v}\mathbf{)}}$ | **Score( µv)** | $\hat{\mathbf{aSE(}\boldsymbol{\mu v}\mathbf{)}}$ | $\hat{\mathbf{mcSE(}\boldsymbol{\mu v}\mathbf{)}}$ |
| S1 | 1.34 | 0.12 | 0.11 | 0.64 | 0.06 | 0.06 |
| S2 | 1.29 | 0.09 | 0.11 | 0.64 | 0.05 | 0.06 |
| S3 | 1.30 | 0.10 | 0.11 | 0.64 | 0.06 | 0.06 |
| S4 | 1.31 | 0.10 | 0.11 | 0.64 | 0.05 | 0.05 |
| S5 | 1.31 | 0.10 | 0.11 | 0.64 | 0.05 | 0.06 |
| S6 | 1.33 | 0.08 | 0.11 | 0.63 | 0.04 | 0.05 |
| S7 | 1.30 | 0.10 | 0.11 | 0.63 | 0.05 | 0.06 |
| S8 | 1.34 | 0.12 | 0.10 | 0.64 | 0.06 | 0.05 |
| S9 | 1.29 | 0.09 | 0.11 | 0.63 | 0.05 | 0.06 |
| S10 | 1.35 | 0.09 | 0.11 | 0.63 | 0.05 | 0.06 |
| S11 | 1.32 | 0.11 | 0.11 | 0.64 | 0.06 | 0.05 |
| S12 | 1.31 | 0.10 | 0.11 | 0.64 | 0.05 | 0.05 |
| S13 | 1.29 | 0.11 | 0.11 | 0.65 | 0.05 | 0.06 |
| S14 | 1.32 | 0.10 | 0.11 | 0.63 | 0.05 | 0.05 |
| S15 | 1.30 | 0.09 | 0.11 | 0.64 | 0.05 | 0.05 |
| S16 | 1.30 | 0.10 | 0.11 | 0.63 | 0.06 | 0.06 |
| S17 | 1.31 | 0.11 | 0.11 | 0.64 | 0.06 | 0.05 |
| S18 | 1.32 | 0.09 | 0.11 | 0.64 | 0.05 | 0.06 |
| S19 | 1.29 | 0.11 | 0.11 | 0.64 | 0.06 | 0.06 |
| S20 | 1.28 | 0.10 | 0.11 | 0.64 | 0.05 | 0.06 |
| **Mean** | **1.31** | **0.10** | **0.11** | **0.64** | **0.05** | **0.06** |
| **SD** | **0.02** | **0.01** | **0.00** | **0.01** | **0.01** | **0.00** |
| **RMS(**$\hat{\boldsymbol{SE}}$**)** | **-** | **0.10** | **0.11** | **-** | **0.05** | **0.06** |

**Table S5.** The obtained $\hat{aSE}$ and $\hat{mcSE}$ of spatial correlation of N2, i.e., the correlation between the estimated N2 and ground truth topography (predefined N2) in the simulated data.

|  | **Cond1** | | | **Cond2** | | |
| --- | --- | --- | --- | --- | --- | --- |
| **Subj_ID** | **Score** | $\hat{\boldsymbol{aSE}}$ | $\hat{\boldsymbol{mcSE}}$ | **Score** | $\hat{\boldsymbol{aSE}}$ | $\hat{\boldsymbol{mcSE}}$ |
| S1 | 1.00 | 0.063 | 0.057 | 1.00 | 0.061 | 0.054 |
| S2 | 0.98 | 0.095 | 0.060 | 1.00 | 0.097 | 0.053 |
| S3 | 1.00 | 0.091 | 0.052 | 1.00 | 0.099 | 0.054 |
| S4 | 1.00 | 0.082 | 0.049 | 1.00 | 0.078 | 0.055 |
| S5 | 0.96 | 0.053 | 0.051 | 1.00 | 0.062 | 0.057 |
| S6 | 0.98 | 0.057 | 0.051 | 1.00 | 0.068 | 0.057 |
| S7 | 1.00 | 0.049 | 0.049 | 1.00 | 0.049 | 0.057 |
| S8 | 0.98 | 0.086 | 0.051 | 1.00 | 0.089 | 0.055 |
| S9 | 1.00 | 0.026 | 0.054 | 1.00 | 0.038 | 0.056 |
| S10 | 1.00 | 0.051 | 0.054 | 1.00 | 0.058 | 0.057 |
| S11 | 1.00 | 0.062 | 0.055 | 1.00 | 0.066 | 0.056 |
| S12 | 1.00 | 0.026 | 0.050 | 1.00 | 0.027 | 0.057 |
| S13 | 0.97 | 0.078 | 0.049 | 1.00 | 0.069 | 0.058 |
| S14 | 0.98 | 0.086 | 0.052 | 0.99 | 0.082 | 0.056 |
| S15 | 1.00 | 0.065 | 0.055 | 1.00 | 0.077 | 0.056 |
| S16 | 0.97 | 0.053 | 0.050 | 1.00 | 0.045 | 0.059 |
| S17 | 0.98 | 0.038 | 0.048 | 0.98 | 0.047 | 0.060 |
| S18 | 1.00 | 0.001 | 0.053 | 1.00 | 0.009 | 0.057 |
| S19 | 0.97 | 0.078 | 0.052 | 1.00 | 0.072 | 0.058 |
| S20 | 1.00 | 0.039 | 0.052 | 1.00 | 0.043 | 0.058 |
| **Mean** | **0.987** | **0.059** | **0.052** | **0.997** | **0.062** | **0.056** |
| **SD** | **0.013** | **0.025** | **0.003** | **0.003** | **0.023** | **0.002** |
| **RMS(**$\hat{\boldsymbol{SE}}$**)** | **-** | **0.064** | **0.052** | **-** | **0.066** | **0.056** |

**Table S6.** The obtained $\hat{aSE}$ and $\hat{mcSE}$ of spatial correlation between the estimated and predefined P3 component in the simulated data.

|  | **Cond1** | | | **Cond2** | | |
| --- | --- | --- | --- | --- | --- | --- |
| **Subj_ID** | **Score** | $\hat{\boldsymbol{aSE}}$ | $\hat{\boldsymbol{mcSE}}$ | **Score** | $\hat{\boldsymbol{aSE}}$ | $\hat{\boldsymbol{mcSE}}$ |
| S1 | 1.00 | 0.059 | 0.040 | 1.00 | 0.060 | 0.041 |
| S2 | 1.00 | 0.000 | 0.040 | 1.00 | 0.000 | 0.042 |
| S3 | 1.00 | 0.001 | 0.040 | 1.00 | 0.046 | 0.041 |
| S4 | 1.00 | 0.039 | 0.041 | 1.00 | 0.042 | 0.040 |
| S5 | 1.00 | 0.070 | 0.041 | 1.00 | 0.063 | 0.041 |
| S6 | 1.00 | 0.004 | 0.042 | 1.00 | 0.025 | 0.039 |
| S7 | 1.00 | 0.000 | 0.040 | 1.00 | 0.001 | 0.041 |
| S8 | 1.00 | 0.082 | 0.039 | 1.00 | 0.082 | 0.040 |
| S9 | 1.00 | 0.029 | 0.040 | 1.00 | 0.029 | 0.042 |
| S10 | 1.00 | 0.029 | 0.041 | 1.00 | 0.029 | 0.041 |
| S11 | 1.00 | 0.071 | 0.041 | 1.00 | 0.056 | 0.039 |
| S12 | 1.00 | 0.000 | 0.041 | 1.00 | 0.000 | 0.040 |
| S13 | 1.00 | 0.045 | 0.041 | 1.00 | 0.036 | 0.041 |
| S14 | 1.00 | 0.001 | 0.040 | 1.00 | 0.018 | 0.040 |
| S15 | 1.00 | 0.028 | 0.042 | 1.00 | 0.040 | 0.040 |
| S16 | 1.00 | 0.084 | 0.041 | 1.00 | 0.073 | 0.041 |
| S17 | 1.00 | 0.028 | 0.041 | 1.00 | 0.028 | 0.040 |
| S18 | 1.00 | 0.028 | 0.041 | 1.00 | 0.039 | 0.040 |
| S19 | 1.00 | 0.030 | 0.041 | 1.00 | 0.055 | 0.041 |
| S20 | 1.00 | 0.000 | 0.042 | 1.00 | 0.001 | 0.040 |
| **Mean** | **0.999** | **0.031** | **0.041** | **0.998** | **0.036** | **0.041** |
| **SD** | **0.000** | **0.029** | **0.001** | **0.000** | **0.024** | **0.001** |
| **RMS(**$\hat{\boldsymbol{SE}}$**)** | **-** | **0.042** | **0.041** | **-** | **0.043** | **0.041** |

**Table S7.** Calculated $\hat{aSE}$ and $\hat{mcSE}$ results from the inner similarity of the N2 component in the estimated time window of the individual subjects.

|  | **Cond1** | | | **Cond2** | | |
| --- | --- | --- | --- | --- | --- | --- |
| **Subj_ID** | **Score_C1** | **aSE_C1** | **mcSE_C1** | **Score_C2** | **aSE_C2** | **mcSE_C2** |
| S1 | 0.85 | 0.009 | 0.011 | 0.86 | 0.009 | 0.012 |
| S2 | 0.84 | 0.011 | 0.012 | 0.86 | 0.011 | 0.012 |
| S3 | 0.83 | 0.010 | 0.011 | 0.83 | 0.011 | 0.012 |
| S4 | 0.85 | 0.010 | 0.011 | 0.86 | 0.010 | 0.012 |
| S5 | 0.82 | 0.010 | 0.011 | 0.85 | 0.010 | 0.012 |
| S6 | 0.82 | 0.011 | 0.012 | 0.85 | 0.010 | 0.012 |
| S7 | 0.83 | 0.015 | 0.011 | 0.85 | 0.021 | 0.012 |
| S8 | 0.83 | 0.011 | 0.011 | 0.88 | 0.013 | 0.012 |
| S9 | 0.84 | 0.010 | 0.012 | 0.84 | 0.010 | 0.011 |
| S10 | 0.83 | 0.011 | 0.011 | 0.83 | 0.014 | 0.011 |
| S11 | 0.84 | 0.009 | 0.011 | 0.86 | 0.012 | 0.012 |
| S12 | 0.85 | 0.011 | 0.011 | 0.86 | 0.011 | 0.011 |
| S13 | 0.83 | 0.011 | 0.011 | 0.85 | 0.009 | 0.011 |
| S14 | 0.82 | 0.010 | 0.011 | 0.85 | 0.012 | 0.011 |
| S15 | 0.85 | 0.013 | 0.011 | 0.88 | 0.011 | 0.011 |
| S16 | 0.85 | 0.009 | 0.011 | 0.86 | 0.010 | 0.011 |
| S17 | 0.84 | 0.010 | 0.011 | 0.86 | 0.010 | 0.012 |
| S18 | 0.84 | 0.011 | 0.011 | 0.86 | 0.011 | 0.011 |
| S19 | 0.86 | 0.009 | 0.012 | 0.85 | 0.009 | 0.011 |
| S20 | 0.83 | 0.013 | 0.011 | 0.85 | 0.011 | 0.011 |
| **Mean** | **0.838** | **0.011** | **0.011** | **0.855** | **0.011** | **0.011** |
| **SD** | **0.012** | **0.002** | **0.000** | **0.012** | **0.003** | **0.000** |
| **RMS(SE)** | **-** | **0.011** | **0.011** | **-** | **0.011** | **0.011** |

**Table S8.** The obtained $\hat{aSE}$ and $\hat{mcSE}$ results from the inner similarity scores of the P3 component in the estimated time window of the individual subjects.

|  | **Cond1** | | | **Cond2** | | |
| --- | --- | --- | --- | --- | --- | --- |
| **Subj_ID** | **Score_C1** | **aSE_C1** | **mcSE_C1** | **Score_C2** | **aSE_C2** | **mcSE_C2** |
| S1 | 0.98 | 0.023 | 0.019 | 0.97 | 0.025 | 0.022 |
| S2 | 0.98 | 0.011 | 0.019 | 0.97 | 0.014 | 0.023 |
| S3 | 0.98 | 0.022 | 0.019 | 0.97 | 0.027 | 0.022 |
| S4 | 0.98 | 0.022 | 0.019 | 0.97 | 0.022 | 0.021 |
| S5 | 0.98 | 0.021 | 0.019 | 0.97 | 0.023 | 0.022 |
| S6 | 0.98 | 0.021 | 0.019 | 0.97 | 0.021 | 0.021 |
| S7 | 0.98 | 0.016 | 0.019 | 0.97 | 0.019 | 0.022 |
| S8 | 0.98 | 0.030 | 0.019 | 0.97 | 0.034 | 0.021 |
| S9 | 0.98 | 0.012 | 0.019 | 0.97 | 0.015 | 0.022 |
| S10 | 0.98 | 0.015 | 0.020 | 0.97 | 0.019 | 0.022 |
| S11 | 0.98 | 0.024 | 0.019 | 0.97 | 0.023 | 0.021 |
| S12 | 0.98 | 0.012 | 0.020 | 0.97 | 0.014 | 0.021 |
| S13 | 0.98 | 0.023 | 0.019 | 0.97 | 0.023 | 0.022 |
| S14 | 0.98 | 0.020 | 0.019 | 0.97 | 0.022 | 0.022 |
| S15 | 0.98 | 0.016 | 0.020 | 0.97 | 0.022 | 0.021 |
| S16 | 0.98 | 0.026 | 0.020 | 0.97 | 0.030 | 0.022 |
| S17 | 0.98 | 0.018 | 0.020 | 0.97 | 0.021 | 0.022 |
| S18 | 0.98 | 0.014 | 0.019 | 0.97 | 0.017 | 0.022 |
| S19 | 0.98 | 0.019 | 0.019 | 0.97 | 0.025 | 0.022 |
| S20 | 0.98 | 0.016 | 0.020 | 0.97 | 0.017 | 0.022 |
| **Mean** | **0.983** | **0.019** | **0.019** | **0.968** | **0.022** | **0.022** |
| **SD** | **0.001** | **0.005** | **0.000** | **0.001** | **0.005** | **0.000** |
| **RMS(SE)** | **-** | **0.020** | **0.019** | **-** | **0.022** | **0.022** |
